# Methylenetetrahydrofolate reductase *C677T* and *A1298C* polymorphisms and gastric cancer susceptibility: an updated meta-analysis

**DOI:** 10.1042/BSR20222553

**Published:** 2023-04-19

**Authors:** Yuwei Wang, Lili Huo, Changqing Yang, Xiaofeng He

**Affiliations:** 1 Department of Digestive internal medicine, Heping Hospital Affiliated to Changzhi Medical College, Shanxi, Changzhi 046000, China; 2 Department of Epidemiology, School of Public Health, Southern Medical University, Guang-dong, Guangzhou 510515, China; 3 Institute of Evidence-Based Medicine, Heping Hospital Affiliated to Changzhi Medical College, Shanxi, Changzhi 046000, China

**Keywords:** A1298C, C677T, gastric cancer, meta-analysis, MTHFR

## Abstract

Widely regarded as one of the most prevalent malignancies worldwide, gastric cancer (GC) is a common clinical condition of the digestive system. Reviewing 14 meta-analyses that evaluated the association between methylenetetrahydrofolate reductase (*MTHFR*) gene polymorphisms and GC risk, we observed inconsistent results, and the credibility of the significant correlation between the statistical results was ignored. With the aim of further exploring the association between *MTHFR C677T* and *A1298C* and the risk of GC, we searched electronic databases, pooling 43 relevant studies and calculating odds ratios (ORs) and corresponding 95% confidence intervals (CIs) for each of the five genetic models. Subgroup and regression analyses were performed to look for sources of heterogeneity and publication bias was assessed by funnel plots. To assess the plausibility of statistically significant associations, we used the FPRP test and the Venice criteria. Overall data analysis showed that *MTHFR C677T* polymorphism was significantly associated with GC risk, especially in Asians, while *MTHFR A1298C* polymorphism was not associated with GC risk. However, in subgroup analysis by hospital-based controls, we found that *MTHFR A1298C* might be a protective factor for GC. After credibility assessment, the statistical association between *MTHFR C677T* and GC susceptibility study was classified as ‘less credible positive result’, while the result of *MTHFR A1298C* was considered unreliable. In summary, the present study strongly suggests that *MTHFR C677T and A1298C* polymorphisms are not significantly associated with the GC risk.

## Introduction

In the last few decades, while the incidence and mortality rates of gastric cancer (GC) have decreased dramatically in many countries [1,2], according to the latest statistics, GC is the fifth most common malignancy in the world, with about 1.1 million new cases in 2020, and is the fourth leading cause of cancer deaths, with about 800,000 deaths [1,2]. The etiology of GC is not fully understood, but multiple factors have been linked to it [3–6], including Helicobacter pylori infection, high intake of nitrites and smoked foods, lifestyle choices, smoking, obesity, radiation, and a genetic predisposition. Remarkably, it has been shown that GC is linked to the expression of various genes involved in folate metabolism, but there is no consensus on the relationship between *MTHFR* gene polymorphisms and GC [7].

Methylenetetrahydrofolate reductase (*MTHFR*), as a core regulatory enzyme in folate metabolism, catalyzes the irreversible conversion of 5,10 methylenetetrahydrofolate (methylene-THF) to 5-methyl-THF, and plays a key role in DNA synthesis, repair and DNA methylation, etc. [8,9]. *MTHFR* has several SNP (single-nucleotide polymorphism) loci, of which the *C677T (rs1801133)* and *A1298C (rs1801131)* loci are two clinically important polymorphic loci. *Rs1801133* is situated in exon 4 and switches Cytosine (C) to Thymine (T) at nucleotide 677, prompting the conversion of alanine into valine at position 222, which has three genotypes, CC, CT and TT [10]. Exon 7 *rs1801131* converts adenine (A) to cytosine (C) at nucleotide 1298, resulting in the mutation of glutamate to alanine, with genotypes AA, AC and CC at this locus [11]. This series of alterations leads to reduced enzyme activity and abnormal genomic DNA methylation, which in turn promotes the development of cancer [12].

In fact, *MTHFR C677T* and *A1298C* gene polymorphisms have been widely studied in various cancers, such as hepatocellular carcinoma [13],colorectal cancer [14], non–Hodgkin’s lymphoma [15], breast cancer [16], etc., while their association with susceptibility to GC has been extensively studied, the findings are still inconclusive [8,9,17–64]. In addition, new original studies have been published in recent years [65–71], but few meta-analyses have been published, and there are problems such as the lack of timely updates, irregular report quality and lack of inclusion in the Chinese literature, so it is necessary to provide a more comprehensive and detailed description of the relationship between *MTHFR* gene polymorphisms and GC susceptibility. As a result of genetic heterogeneity, it is also necessary to explore the source of heterogeneity using subgroup analysis and sensitivity analysis. Based on a meta-analysis of existing case–control studies and cohort studies, this study further examined whether the *C677T* and *A1298C* polymorphisms of the *MTHFR* gene are associated with GC risk, providing a reference for population-based gastric cancer risk assessment and prevention and control.

## Results

### Description of included studies

**Figure 1:**
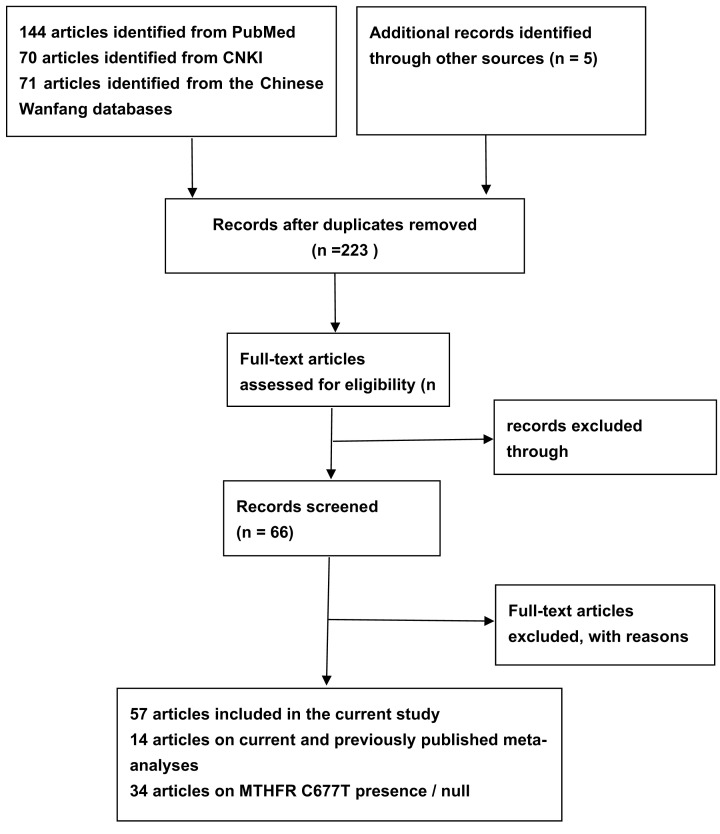
Flow diagram of the literature search

Figure 1 shows a more detailed document search process. As you can see, our search yielded 223 articles, and 43 studies met our requirements based on inclusion and exclusion criteria (comprising 11,146 GC cases and 15,688 healthy or non-cancerous controls) [8,30–71] with publication years between 2001 and 2021. Among them, 34 studies investigated the relationship between *MTHFR C677T* and GC (11,146 GC cases and 15,688 controls), and 19 studies examined *MTHFR A1298C* (3920 patients and 5920 controls). Twenty-nine of these studies were dedicated to Asians, 14 to Caucasians, and none to Indians or mixed populations. In addition, we conducted a quality assessment of the included literature and the results showed that 26 studies with a high-quality and 13 medium-quality studies and 4 low-quality studies studied the association between *MTHFR C677T* and risk of GC; In contrast, of the studies on *MTHFR A1298C* and the risk of GC, 12, 6 and 1 rated as high, medium and low quality, respectively. Detailed results of genotype frequencies, HWE tests and quality scores of *MTHFR C677T* and *A1298C* in relation to GC risk are shown in Supplementary Table S2.

### Meta-analysis results

#### MTHFR C677T (rs1801133)

Based on the results of the overall analysis, we can conclude that *MTHFR C677T* increases the risk of GC. The results of the individual gene models are as follows: TT vs. CC: OR = 1.318, 95% CI = 1.146–1.515; CT vs. CC: OR = 1.128, 95% CI = 1.017–1.252; TT vs. (CC+CT) : OR = 1.163, 95% CI = 1.090–1.241; CT+TT vs. CC: OR = 1.174,95% CI = 1.056–1.306; T vs. C: OR = 1.144, 95% CI = 1.064–1.230, Table 1). In the next subgroup analysis, we found a significant association between *MTHFR C677T polymorphism* and GC in Asians (TT vs. CC: OR = 1.363, 95% CI = 1.143–1.626; CT vs. CC: OR = 1.146, 95% CI = 1.012–1.299; TT vs. CC+CT: OR = 1.140, 95% CI = 1.098–1.401; CT+TT vs. CC: OR = 1.212, 95% CI = 1.064–1.380; T vs C: OR = 1.176, 95% CI = 1.075–1.286) and Caucasians (TT vs. CC: OR = 1.244, 95% CI = 1.058–1.46, Table 1).

**Table 1:** Meta-analysis of the association of *MTHFR C677T* polymorphism with risk of gastric cancer

Variable	*n* (Cases/Controls)		TT vs. CC		CT vs.CC		TT vs. CC+CT		CT+TT vs. CC		T vs. C	
			OR (95% CI)	Ph/*I*^2^ (%)	OR (95% CI)	Ph/*I*^2^ (%)	OR (95% CI)	Ph/*I*^2^ (%)	OR (95% CI)	Ph/*I*^2^ (%)	OR (95% CI)	Ph/*I*^2^ (%)
Overall	43 (11148/15688)	REM	1.318 (1.146– 1.515)	0.000/65.3	1.128 (1.017–1.252)	0.000/63.9	1.211 (1.097– 1.336)	0.000/46.8	1.174 (1.056–1.306)	0.000/70.0	1.144 (1.064– 1.230)	0.000/71.0
		FEM					1.163 (1.090– 1.241)	0.000/46.8				
Ethnicity												
Asian	29 (8885/11389)	REM	1.363 (1.143–1.626)	0.000/72.1	1.146 (1.012–1.299)	0.000/67.3	1.240 (1.098–1.401)	0.000/55.7	1.212 (1.064–1.380)	0.000/73.6	1.176 (1.075–1.286)	0.000/75.6
Caucasian	14 (2263/4299)	REM	1.243 (1.004–1.538)	0.089/35.8	1.088 (0.895–1.323)	0.005/56.7	1.137 (0.968–1.335)	0.277/16.1	1.098 (0.907–1.328)	0.002/61.0	1.078 (0.950– 1.222)	0.004/57.0
		FEM	1.244 (1.058–1.462)	0.089/35.8			1.118 (0.970–1.288)	0.277/16.1				
Source of control												
HB	30 (5918/9297)	REM	1.322 (1.105–1.582)	0.000/64.2	1.197 (1.054–1.360)	0.000/58.0	1.181 (1.039–1.342)	0.003/46.8	1.225 (1.074–1.397)	0.000/65.9	1.158 (1.057–1.269)	0.000/68.4
		FEM					1.161 (1.066–1.265)	0.003/46.8				
PB	12 (5154/6300)	REM	1.321 (1.046–1.668)	0.000/70.2	1.039 (0.882–1.225)	0.001/65.8	1.270 (1.075–1.501)	0.011/55.1	1.118 (0.941–1.327)	0.000/72.5	1.140 (1.010–1.287)	0.000/75.3
Type of control												
Healthy	19 (4661/6829)	REM	1.398 (1.113–1.756)	0.000/70.0	1.158 (0.991–1.354)	0.000/64.8	1.230 (1.050–1.441)	0.005/51.3	1.208 (1.205–1.425)	0.000/72.4	1.165 (1.036–1.311)	0.000/74.7
Non–gastric cancer	24 (6487/8859)	REM	1.265 (1.058–1.511)	0.000/61.3	1.105 (0.959–1.274)	0.000/63.6	1.200 (1.055–1.366)	0.009/45.1	1.149 (0.997– 1.324)	0.000/68.4	1.129 (1.027–1.240)	0.000/68.2
		FEM					1.154 (1.060–1.257)	0.009/45.1				
HWE and Quality score > 12												
Overall	23 (6687/9799)	REM	1.423 (1.156–1.753)	0.000/73.9	1.194 (1.019–1.399)	0.000/74.4	1.254 (1.096–1.436)	0.001/53.2	1.260 (1.071–1.482)	0.000/78.4	1.197 (1.076–1.332)	0.000/78.2
Ethnicity												
Asian	15 (5070/6545)	REM	1.495 (1.133–1.972)	0.000/79.4	1.230 (0.996–1.519)	0.000/78.7	1.299 (1.097–1.538)	0.002/58.8	1.319 (1.060–1.641)	0.000/82.4	1.244 (1.080–1.434)	0.000/82.6
Caucasian	8 (1617/3254)	REM	1.314 (0.966–1.786)	0.026/56.1	1.145 (0.895–1.464)	0.006/64.5	1.167 (0.920–1.481)	0.085/44.0	1.173 (0.917–1.499)	0.002/68.3	1.116 (0.948–1.314)	0.004/66.3
		FEM					1.107 (0.933–1.312)	0.085/44.0				
Source of control												
HB	14 (2753/5031)	REM	1.467 (1.070–2.013)	0.000/75.5	1.280 (1.024–1.599)	0.000/73.2	1.225 (0.995–1.507)	0.004/57.4	1.325 (1.050–1.672)	0.000/78.0	1.211 (1.035–1.417)	0.000/78.8
PB	9 (3934/4768)	REM	1.379 (1.040–1.828)	0.000/72.2	1.092 (0.869–1.371)	0.000/74.2	1.295 (1.078–1.555)	0.036/51.6	1.184 (0.938–1.494)	0.000/78.3	1.181 (1.014–1.376)	0.000/78.3
Type of control												
Healthy	8 (1538/3203)	REM	1.808 (1.252–2.610)	0.001/70.4	1.308 (0.968–1.767)	0.000/76.0	1.463 (1.195–1.791)	0.221/26.1	1.413 (1.047–1.909)	0.000/78.4	1.319 (1.093–1.591)	0.000/75.6
		FEM					1.432 (1.207–1.697)	0.221/26.1				
Non-gastric cancer	15 (5149/6596)	REM	1.259 (0.990–1.600)	0.000/71.5	1.146 (0.947–1.386)	0.000/73.8	1.157 (0.984–1.361)	0.006/54.6	1.192 (0.981–1.447)	0.000/77.9	1.138 (1.002–1.291)	0.000/77.4
Egger’s test												
*P*_E_			0.012		0.003		0.171		0.002		0.018	

Abbreviations: FEM, fixed effects model; HB, hospital-based studies; PB, population-based studies; REM, random effects model.

**Figure 2:**
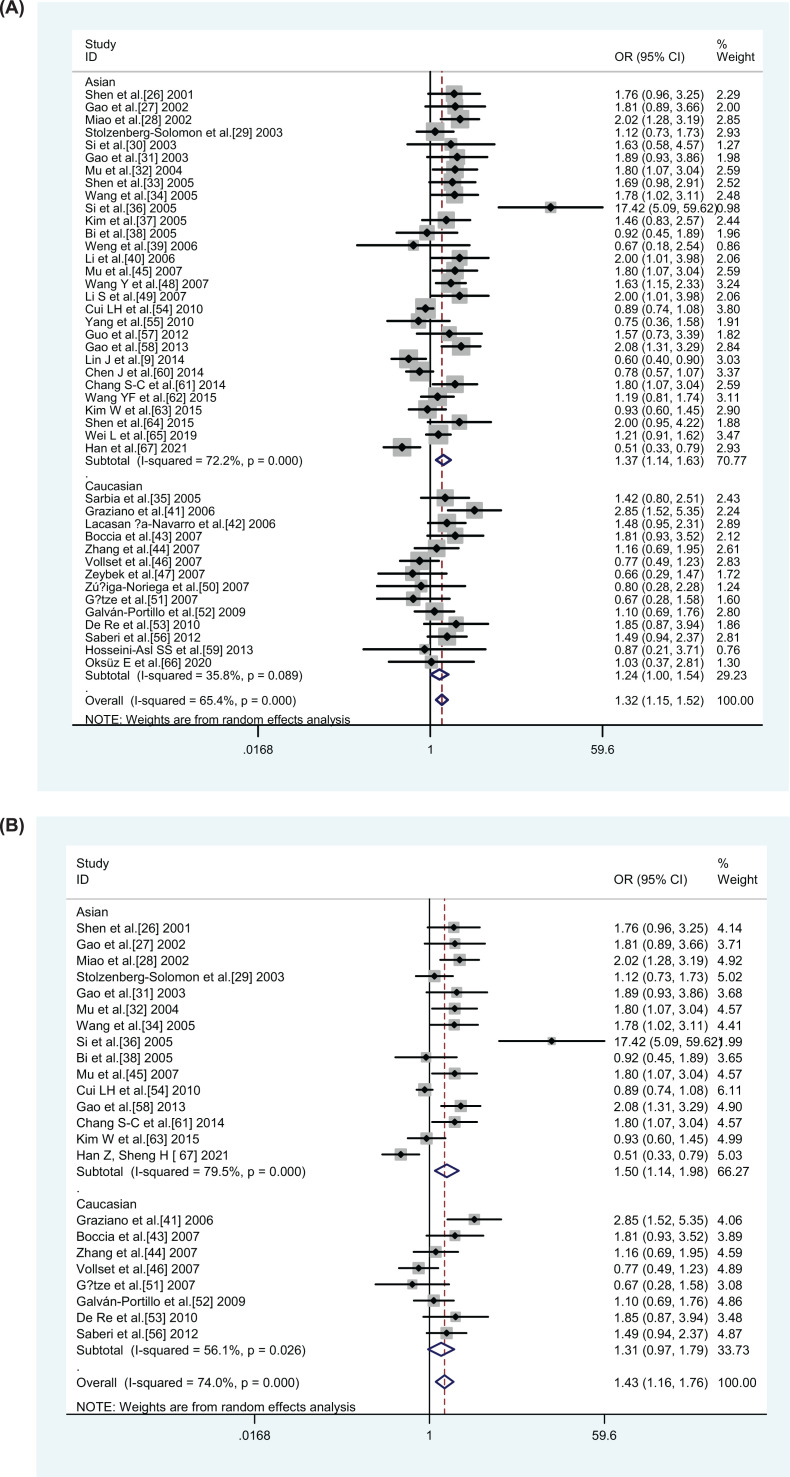
Racial subgroup analysis of *MTHFR C677T* polymorphism with GC risk correlation forest graph (TT vs. CC) (**A**) Overall analysis and (**B**) sensitivity analysis.

In subgroup analysis according to control types, the results showed a positive association in hospital-based studies (TT vs. CC: OR = 1.322, 95% CI = 1.105–1.582; CT vs. CC: OR = 1.197, 95% CI = 1.054–1.360; TT vs. (CC + CT): OR = 1.161, 95% CI = 1.066–1.265; (CT+TT) vs. CC: OR = 1.225, 95% CI = 1.074–1.397; T vs. C: OR = 1.158, 95% CI = 1.057–1.269) and population-based studies (TT vs CC: OR = 1.321, 95% CI = 1.046–1.668; TT vs. CC+CT: OR = 1.270, 95% CI = 1.075–1.501; T vs C: OR = 1.140, 95% CI = 1.010–1.287) indicating that *MTHFR C677T polymorphism* increased the risk of GC. By undertaking a detailed reading of all included studies, we performed further peptide variable analysis for tumor location and differentiation type (Supplementary Table S3 shows the detailed gene distribution for subgroup analysis) and found that *MTHFRC677T polymorphism* added to the susceptibility of patients with cardia cancer (T vs. C: OR = 1.142, 95% CI = 1.022–1.275), while no correlation was observed in non-cardia cancer studies. Moreover, similar positive results were also found in the subgroup analysis for both intestinal type (TT vs. CC: OR = 1.732, 95% CI = 1.211–2.475; TT vs. CC+CT: OR = 1.410, 95% CI = 1.027–1.935, Table 2a) and diffuse type (TT vs. CC: OR = 1.478, 95% CI = 1.023–2.135, Table 2b). The results of the forest plot for the racial subgroup analysis of the *MTHFR C677T* polymorphism associated with GC risk are shown in Figure 2A (TT vs. CC, overall analysis).

**Table 2a:** Meta-analysis of the association of *MTHFR C677T (rs1801133)* polymorphism with risk of gastric cancer

Variable		TT vs. CC		CT vs. CC		TT vs. CC + CT		CT+TT vs. CC		T vs. C	
		OR (95% CI)	Ph/*I*^2^ (%)	OR (95% CI)	Ph*/I*^2^ (%)	OR (95% CI)	Ph/*I*^2^ (%)	OR (95% CI)	Ph/*I*^2^ (%)	OR (95% CI)	Ph/*I*^2^ (%)
Subgroupanalysis											
Tumor location											
Cardia 9(995/2925)	REM	1.227 (0.822–1.832)	0.047/49.0	1.175 (0.823–1.676)	0.000/73.3	1.201 (0.837–1.725)	0.038/51.0	1.186 (0.869–1.617)	0.001/68.5	1.146 (0.972–1.351)	0.058/46.8
	FEM	1.215 (0.951–1.553)	0.047/49.0							1.142 (1.022–1.275)	0.058/46.8
Non-cardia 9(1589/2925)	REM	1.275 (0.907–1.792)	0.025/54.3	1.172 (0.926–1.483)	0.014/58.4	1.174 (0.915–1.507)	0.160/32.3	1.214 (0.951– 1.549)	0.003/65.4	1.172 (0.985–1.396)	0.003/65.9
	FEM					1.103 (0.912–1.333)	0.160/32.3				
Histologic subtype											
Intestinal type 5(403/1568)	REM	1.735 (1.210–2.490)	0.417/0.0	1.215 (0.801–1.841)	0.045/58.9	1.416 (1.030–1.945)	0.828/0.0	1.272 (0.840–1.927)	0.028/63.1	1.229 (0.959–1.575)	0.090/50.2
	FEM	1.732 (1.211–2.475)	0.417/0.0			1.410 (1.027–1.935)	0.828/0.0				
Diffuse type 5(326/1568)	REM	1.473 (0.943–2.301)	0.291/19.4	1.022 (0.601–1.739)	0.010/69.8	1.337 (0.964–1.854)	0.805/0.0	1.059 (0.626–1.792)	0.005/72.8	1.090 (0.783–1.517)	0.022/65.1
	FEM	1.478 (1.023–2.135)	0.291/19.4			1.325 (0.957–1.834)	0.805/0.0				

**Table 2b:** Meta-analysis of the association of MTHFR A1298C (rs1801131) polymorphism with risk of gastric cancer

Variable		CC vs. AA		CC vs. AC		CC vs. AA+AC		AC+CC vs. AA		C vs. A	
		OR (95% CI)	Ph/*I*^2^ (%)	OR (95% CI)	Ph/*I*^2^ (%)	OR (95% CI)	Ph/*I*^2^ (%)	OR (95% CI)	Ph/*I*^2^ (%)	OR (95% CI)	Ph/*I*^2^ (%)
Subgroupanalysis											
Tumor Location											
Cardia 4 (387/913)	REM	1.300 (0.319–5.302)	0.178/39.1	0.922 (0.697–1.219)	0.907/0.0	1.285 (0.326–5.069)	0.186/37.7	0.930 (0.708–1.223)	0.754/0.0	0.956 (0.750–1.219)	0.516/0.0
	FEM	1.132 (0.470–2.727)	0.178/39.1	0.921 (0.697–1.218)	0.907/0.0	1.140 (0.477–2.726)	0.186/37.7	0.928 (0.707–1.219)	0.754/0.0	0.950 (0.746–1.211)	0.516/0.0
Non-cardia 4 (430/913)	REM	0.883 (0.377–2.067)	0.340/7.3	1.132 (0.766–1.675)	0.099/52.2	0.834 (0.380–1.828)	0.424/0.0	1.086 (0.732–1.610)	0.085/54.7	1.027 (0.736–1.434)	0.097/52.5
	FEM	0.823 (0.383–1.771)	0.340/7.3			0.757 (0.354–1.621)	0.424/0.0				

Abbreviations: FEM, fixed effects model; REM, random effects model.

#### MTHFR A1298C (rs1801131)

**Figure 3:**
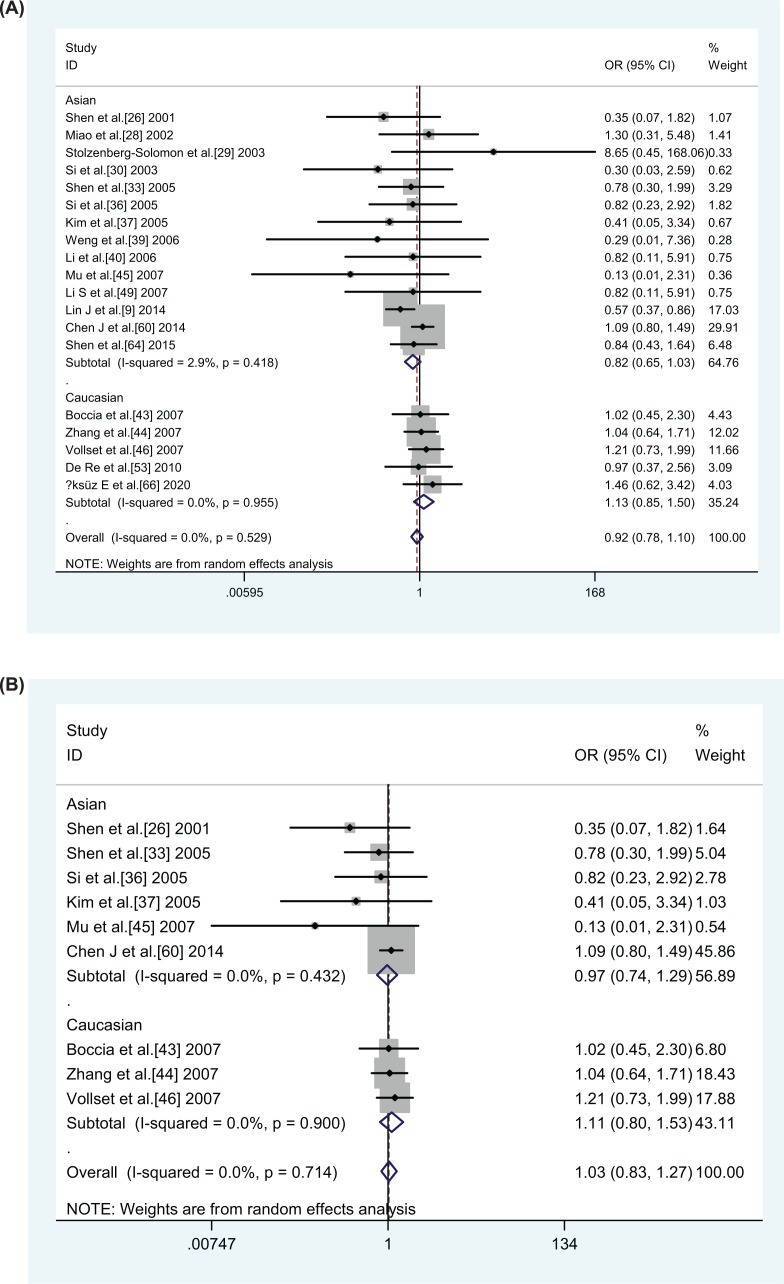
Racial subgroup analysis of *MTHFR A1298C* polymorphism with GC risk correlation forest graph (CC vs. AA+AC) (**A**) Overall analysis. (**B**) sensitivity analysis.

In terms of overall data, *MTHFR A1298C* did not be associated (AA vs. CC: OR = 0.935, 95% CI = 0.784–1.115; AC vs. CC: OR = 1.023, 95% CI = 0.935–1.119; (AA+AC) vs. CC: OR = 0.908, 95% CI = 0.768–1.075; AA vs. (AC+CC): OR = 1.005, 95% CI = 0.922–1.097; C vs. A: OR = 0.987, 95% CI = 0.921–1.058, Table 3) with GC susceptibility. Also, no correlations were observed in subgroup analyses based on ethnicity, type of control and population-based studies. What is noteworthy, however, is that CC vs. AA model (OR = 0.755, 95% CI = 0.574–0.991 and CC vs. AA+AC model (OR = 0.741, 95% CI = 0.571–0.963, Table 3) revealed a negative association between the *MTHFR A1298C* polymorphism and increased GC susceptibility, which was found in the hospital-based subgroup analysis emerged. Furthermore, in further tumor location-based subgroup analysis, no correlation was observed (Table 3). Figure 3A shows a forest plot of the ethnic subgroup analysis of the *MTHFR A1298C* polymorphism in relation to GC risk (CC vs. AA+AC, overall analysis).

**Table 3:** Meta-analysis of the association of *MTHFR A1298C (rs1801131)* polymorphism with risk of gastric cancer

Variable	*n* (Cases/Controls)		CC vs. AA		AC vs. AA		CC vs. AA+AC		AC +CC vs. AA		C vs. A	
			OR (95% CI)	Ph/*I*^2^ (%)	OR (95% CI)	Ph/*I*^2^ (%)	OR (95% CI)	Ph/*I*^2^ (%)	OR (95% CI)	Ph/*I*^2^ (%)	OR (95% CI)	Ph/*I*^2^ (%)
Overall	19 (3920/5988)	REM	0.943 (0.734–1.210)	0.132/27.3	1.019 (0.908–1.142)	0.100/30.7	0.924 (0.779–1.096)	0.529/0.0	0.999 (0.883–1.129)	0.025/42.8	0.980 (0.885–1.084)	0.019/44.7
		FEM	0.935 (0.784–1.115)	0.132/27.3	1.023 (0.935–1.119)	0.100/30.7	0.908 (0.768–1.075)	0.529/0.0	1.005 (0.922–1.097)	0.025/42.8	0.987 (0.921–1.058)	0.019/44.7
Ethnicity												
Asian	14 (3156/4205)	REM	0.781 (0.583–1.048)	0.294/14.6	0.979 (0.883–1.086)	0.792/0.0	0.817 (0.650–1.026)	0.418/2.9	0.953 (0.862–1.054)	0.487/0.0	0.928 (0.841–1.025)	0.217/21.9
		FEM	0.806 (0.648–1.002)	0.294/14.6	0.979 (0.883–1.085)	0.792/0.0	0.814 (0.662–1.001)	0.418/2.9	0.953 (0.862–1.053)	0.487/0.0	0.938 (0.865–1.017)	0.217/21.9
Caucasian	5 (764/1783)	REM	1.261 (0.898–1.770)	0.318/15.1	1.245 (0.846–1.832)	0.006/72.4	1.128 (0.846–1.504)	0.995/0.0	1.246 (0.862–1.800)	0.005/72.8	1.153 (0.914–1.455)	0.033/61.9
		FEM	1.251 (0.926–1.689)	0.318/15.1			1.127 (0.846–1.502)	0.995/0.0				
Source of control												
HB	12 (1644/2649)	REM	0.828 (0.578–1.185)	0.228/22.0	0.974 (0.819–1.158)	0.157/29.5	0.753 (0.578–0.982)	0.757/0.0	0.949 (0.791–1.140)	0.072/40.3	0.930 (0.804–1.076)	0.080/39.1
		FEM	0.755 (0.574–0.991)	0.228/22.0	0.959 (0.834–1.102)	0.157/29.5	0.741 (0.571–0.963)	0.757/0.0	0.921 (0.805–1.053)	0.072/40.3	0.901 (0.810–1.003)	0.080/39.1
PB	7 (2276/3339)	REM	1.111 (0.854–1.446)	0.369/7.8	1.069 (0.921–1.241)	0.182/32.3	1.070 (0.855–1.338)	0.486/0.0	1.063 (0.911–1.241)	0.124/40.2	1.046 (0.925–1.182)	0.145/37.2
		FEM	1.097 (0.869–1.384)	0.369/7.8	1.072 (0.952–1.206)	0.182/32.3	1.057 (0.847–1.319)	0.486/0.0	1.072 (0.956–1.201)	0.124/40.2	1.054 (0.963–1.154)	0.145/37.2
Type of control												
Healthy	9 (2118/3055)	REM	0.925 (0.648–1.321)	0.042/50.0	0.979 (0.807–1.188)	0.040/50.4	0.896 (0.705–1.140)	0.286/17.6	0.962 (0.782–1.182)	0.010/60.3	0.948 (0.804–1.117)	0.006/62.7
		FEM					0.888 (0.732–1.078)	0.286/17.6				
Non-gastric cancer	10 (1522/2933)	REM	1.065 (0.746–1.521)	0.509/0.0	1.087 (0.952–1.242)	0.507/0.0	0.983 (0.696–1.388)	0.633/0.0	1.076 (0.943–1.227)	0.416/2.5	1.054 (0.945–1.176)	0.447/0.0
		FEM	1.046 (0.740–1.480)	0.509/0.0	1.087 (0.952–1.240)	0.507/0.0	0.974 (0.695–1.365)	0.633/0.0	1.077 (0.947–1.226)	0.416/2.5	1.052 (0.943–1.174)	0.447/0.0
HWE and quality score > 12												
Overall	9 (2232/3462)	REM	1.063 (0.851–1.327)	0.564/0.0	1.045 (0.918–1.190)	0.310/14.8	1.028 (0.832–1.271)	0.714/0.0	1.029 (0.898–1.180)	0.212/26.0	1.014 (0.911–1.130)	0.230/24.0
		FEM	1.034 (0.830–1.289)	0.564/0.0	1.049 (0.934–1.178)	0.310/14.8	1.007 (0.816–1.242)	0.714/0.0	1.041 (0.931–1.163)	0.212/26.0	1.026 (0.940–1.120)	0.230/24.0
Ethnicity												
Asian	6 (1935/2194)	REM	0.956 (0.685–1.333)	0.396/3.2	1.010 (0.877–1.164)	0.467/0.0	0.973 (0.735–1.289)	0.432/0.0	0.988 (0.853–1.145)	0.365/8.0	0.967 (0.846–1.106)	0.299/17.7
		FEM	0.949 (0.711–1.266)	0.396/3.2	1.010 (0.877–1.163)	0.467/0.0	0.941 (0.715–1.240)	0.432/0.0	0.994 (0.867–1.139)	0.365/8.0	0.986 (0.884–1.100)	0.299/17.7
Caucasian	3 (637/1268)	REM	1.172 (0.836–1.642)	0.598/0.0	1.121 (0.835–1.505)	0.138/49.6	1.105 (0.801–1.526)	0.900/0.0	1.128 (0.847–1.501)	0.128/51.4	1.097 (0.911–1.320)	0.217/34.6
		FEM	1.167 (0.832–1.636)	0.598/0.0	1.134 (0.926–1.388)	0.138/49.6	1.104 (0.800–1.525)	0.900/0.0	1.141 (0.941–1.383)	0.128/51.4	1.101 (0.952–1.274)	0.217/34.6
Source of control												
HB	3 (411/521)	REM	0.811 (0.424–1.552)	0.512/0.0	0.956 (0.719–1.272)	0.968/0.0	0.826 (0.439–1.554)	0.519/0.0	0.933 (0.708–1.228)	0.917/0.0	0.928 (0.740–1.164)	0.814/0.0
		FEM	0.797 (0.418–1.521)	0.512/0.0	0.956 (0.719–1.272)	0.968/0.0	0.812 (0.433–1.525)	0.519/0.0	0.933 (0.708–1.228)	0.917/0.0	0.928 (0.740–1.164)	0.814/0.0
PB	6 (1951/2941)	REM	1.102 (0.870–1.395)	0.460/0.0	1.062 (0.890–1.267)	0.116/43.4	1.057 (0.844–1.323)	0.613/0.0	1.049 (0.875–1.257)	0.078/49.5	1.029 (0.894–1.183)	0.102/45.6
		FEM	1.071 (0.848–1.354)	0.460/0.0	1.068 (0.941–1.212)	0.116/43.4	1.035 (0.828–1.293)	0.613/0.0	1.063 (0.942–1.201)	0.078/49.5	1.044 (0.950–1.148)	0.102/45.6
Type of control												
Healthy	4 (1376/1670)	REM	1.051 (0.801–1.379)	0.475/0.0	0.972 (0.832–1.134)	0.714/0.0	1.048 (0.810–1.356)	0.522/0.0	0.975 (0.840–1.130)	0.523/0.0	0.985 (0.874–1.111)	0.365/5.7
		FEM	1.025 (0.783–1.341)	0.475/0.0	0.971 (0.832–1.134)	0.714/0.0	1.026 (0.795–1.324)	0.522/0.0	0.974 (0.840–1.129)	0.523/0.0	0.989 (0.883–1.107)	0.365/5.7
Non-gastric cancer	5 (986/1792)	REM	1.063 (0.707–1.600)	0.376/5.3	1.133 (0.912–1.408)	0.210/31.7	0.988 (0.681–1.433)	0.543/0.0	1.101 (0.878–1.380)	0.148/41.0	1.055 (0.880–1.264)	0.179/36.4
		FEM	1.054 (0.719–1.543)	0.376/5.3	1.158 (0.972–1.379)	0.210/31.7	0.967 (0.668–1.400)	0.543/0.0	1.135 (0.959–1.344)	0.148/41.0	1.085 (0.944–1.246)	0.179/36.4
Egger's test												
*P_E_*			0.485		0.824		0.337		0.988		0.660	

Abbreviations: FEM, fixed effects model; HB, hospital-based studies; PB, population-based studies; REM, random effects model.

### Heterogeneity and sensitivity analyses

The research illustrated that heterogeneity emerged in the overall and several subgroup analyses. Potential factors that could be sources of heterogeneity, such as race, sample source, control type, match type, quality score and HWE were considered, and we used meta-regression analysis for further exploration. The results showed that for *MTHFR C677T*, no covariates were identified as possible causes of between-study variation, while quality score (CC vs. AA: *P*=0.029; C vs. A: *P*=0.047) and sample source (CC vs. AA: *P*=0.047; C vs. A: *P*=0.044) were the causes of the *MTHFR A1298C* polymorphism and GC risk source of heterogeneity between.

A sensitivity analysis of the included studies was performed in this meta-analysis to assess the stability of the studies. To begin with, the included literature was removed one by one, and the OR values of the remaining literature were calculated, and the results of the sensitivity analysis were in line with the original meta-analysis, which suggesting little variation in the quality of included studies and more stable results of the present study (graphics not shown). After that the combined OR in the overall study did not seem to be significantly affected when only high-quality studies, HWE, and matched studies were included. Yet, in the subgroup analysis, for the *MTHFR C677T* polymorphism, the results of the sensitivity analysis showed variability with the original meta-analysis: Caucasians (TT vs. CC: OR = 1.314, 95% CI = 0.966–1.786); hospital-based studies (TT vs. CC: OR = 1.467, 95% CI = 1.070–2.013; CT vs. CC: OR = 1.280, 95% CI = 1.024–1.599; CT+TT vs. CC: OR = 1.325, 95% CI = 1.050–1.672; T VS C: OR = 1.211, 95% CI = 1.035–1.417); Healthy controls (TT vs. CC: OR = 1.467, 95% CI = 1.070–2.013; CT vs. CC: OR = 1.280, 95% CI = 1.024–1.599; CT+TT vs. CC: OR = 1.325, 95% CI = 1.050–1.672; T vs. C: OR = 1.211, 95% CI = 1.035–1.417); non-cancer control (TT vs. CC: OR = 1.259, 95% CI = 0.990–1.600; T vs. C: OR = 1.138, 95% CI = 1.002–1.291). The otherwise reduced risk of GC in a healthy population disappeared for the *MTHFRA1298C* polymorphism when we included only high quality and HWE studies in the control group. Above results of sensitivity analysis remind us that further studies are needed to include more high quality and HWE-compliant articles in the future. The results of the forest plot of *MTHFR C677T* and *A1298C* polymorphisms and susceptibility to GC after sensitivity analysis are shown in Figures 2B and 3B.

### Publication bias

**Figure 4:**
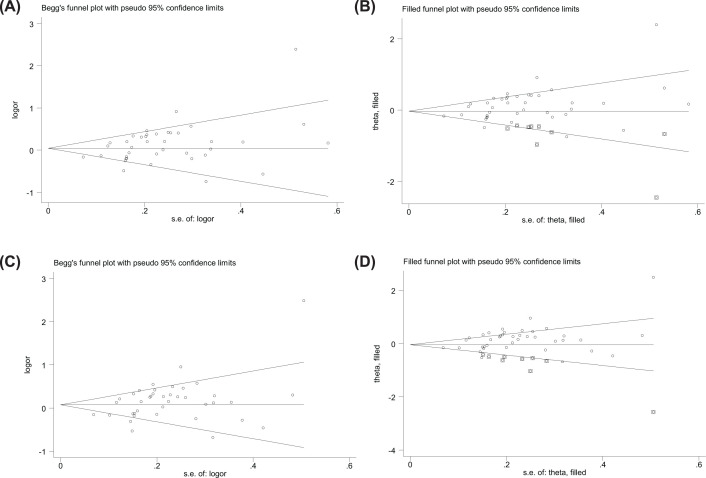
Funnel plots of the relationship between MTHFR C677T polymorphism and susceptibility to GC Assessment of publication bias between MTHFR C677T polymorphism and susceptibility to gastric cancer in the general population by Begg funnel plot (CT vs. CC; CT+TT vs. CC) [(**A**) CT vs. CC; (**B**) CT vs. CC adjusted for publication bias using a nonparametric ‘trim and fill’ approach; (**C**) CT+TT vs. CC; (**D**) CT+TT vs. CC adjusted for publication bias using a nonparametric ‘trim and fill’ approach].

Begg’s funnel plot and Egger’s test indicated publication bias between the *MTHFR C677T* polymorphism and GC risk study results, and the absence of *MTHFR A1298C polymorphism*, as detailed below: funnel plots showed asymmetric distribution of included studies and individual studies outside the confidence interval, see Figure 4, indicating possible publication bias; Egger test (CT vs. CC: *P*=0.003; CT+TT vs. CC: *P*=0.002; Table 1), publication bias was present. We then adjusted for publication bias using a nonparametric ‘trim and fill’ approach, which indicated that we would need to add one and five articles to the CT vs. CC and CT+TT vs. CC models, respectively, in the future (Figure 4) and the publication offset has not affected the results.

### Credibility of the identified genetic associations

The credibility of this meta-analysis was evaluated in terms of the FPRP, Bayesian false discovery probability (BFDP) and Venice criteria. We categorized statistically significant associations as ‘high confidence’ [72] when the following conditions were met. (1) statistically significant associations were observed in at least two genetic models, i.e., *P*-values for the Z-test were <0.05, (2) FPRP was <0.2 and BFDP <0.8, (3) statistical power > 0.8 and (4) *I-*square < 50%. When the following conditions were met, a lower threshold of ‘less plausible certainty’ was taken into account. (1) at least one genetic model had a *P*-value of 0.05.; (2) statistical power was between 50% and 79%, or FPRP > 0.2 or *I*-square > 50%. Otherwise, the association was categorized as ‘null’ or ‘negative’. Statistically significant associations among *MTHFR* polymorphisms and GC susceptibility studies were categorized as ‘under-confident positive results’ after confidence assessment, while statistically significant associations were rated as ‘null’ or ‘negative’ toward the *MTHFR A1298C* polymorphism, in the current meta-analysis. Table 4 describe more details of the credibility assessment.

**Table 4:** Credibility of the current meta-analysis

Variables	Model	OR (95% CI)	*I*^2^ (%)	Statistical power	Credibility	
					Prior probability of 0.001	
					FPRP	BFDP
*MTHFR C677T*						
Overall	TT vs.CC	1.318 (1.146–1.515)	65.3	0.966	0.096	0.825
	CT vs.CC	1.128 (1.017–1.252)	63.9	1.000	0.959	0.999
	TT vs. CC+CT	1.163 (1.090–1.241)	46.8	1.000	0.005	0.350
	CT+TT vs. CC	1.174 (1.056–1.306)	70.0	1.000	0.760	0.993
	T vs. C	1.144 (1.064–1.230)	71.0	1.000	0.215	0.954
Asian	TT vs.CC	1.363 (1.143–1.626)	72.1	0.856	0.404	0.952
	CT vs.CC	1.146 (1.012–1.299)	67.3	1.000	0.971	0.999
	TT vs. CC+CT	1.240 (1.098–1.401)	55.7	0.999	0.356	0.962
	CT+TT vs. CC	1.212 (1.064–1.380)	73.6	0.999	0.787	0.993
	T vs. C	1.176 (1.075–1.286)	75.6	1.000	0.275	0.959
Caucasian	TT vs.CC	1.244 (1.058–1.462)	35.8	0.988	0.890	0.995
HB	TT vs.CC	1.322 (1.105–1.582)	64.2	0.916	0.716	0.985
	CT vs.CC	1.197 (1.054–1.360)	58.0	1.000	0.852	0.995
	TT vs. CC+CT	1.161 (1.066–1.265)	46.8	1.000	0.393	0.975
	CT+TT vs. CC	1.225 (1.074–1.397)	65.9	0.999	0.712	0.989
	T vs. C	1.158 (1.057–1.269)	68.4	1.000	0.627	0.989
PB	TT vs.CC	1.321 (1.046–1.668)	70.2	0.857	0.957	0.997
	TT vs. CC+CT	1.270 (1.075–1.501)	55.1	0.975	0.838	0.993
	T vs. C	1.140 (1.010–1.287)	75.3	1.000	0.972	0.989
Healthy	TT vs.CC	1.398 (1.113–1.756)	70.0	0.728	0.845	0.989
	TT vs. CC+CT	1.230 (1.050–1.441)	51.3	0.993	0.913	0.996
	CT+TT vs. CC	1.208 (1.205–1.425)	72.4	0.995	0.962	0.998
	T vs. C	1.165 (1.036–1.311)	74.7	1.000	0.918	0.997
Non-gastric cancer	TT vs. CC	1.165 (1.036–1.311)	61.3	1.000	0.918	1.000
	TT vs. CC+CT	1.154 (1.060–1.257)	45.1	1.000	0.506	0.984
HWE and Quality score > 12	T vs. C	1.129 (1.027–1.240)	68.2	1.000	0.918	0.998
Overall	TT vs.CC	1.423 (1.156–1.753)	73.9	0.690	0.570	0.964
	CT vs.CC	1.194 (1.019–1.399)	74.4	0.998	0.966	0.998
	TT vs. CC+CT	1.254 (1.096–1.436)	53.2	0.995	0.516	0.9777
	CT+TT vs. CC	1.260 (1.071–1.482)	78.4	0.982	0.842	0.993
	T vs. C	1.197 (1.076–1.332)	78.2	1.000	0.493	0.979
Asian	TT vs.CC	1.495 (1.133–1.972)	79.4	0.509	0.894	0.989
	TT vs. CC+CT	1.299 (1.097–1.538)	58.8	0.953	0.716	0.986
	CT+TT vs. CC	1.319 (1.060–1.641)	82.4	0.876	0.937	0.996
	T vs. C	1.244 (1.080–1.434)	82.6	0.995	0.723	0.989
HB	TT vs.CC	1.467 (1.070–2.013)	75.5	0.555	0.969	0.996
	CT vs.CC	1.280 (1.024–1.599)	73.2	0.919	0.970	0.998
	CT+TT vs. CC	1.325 (1.050–1.672)	78.0	0.852	0.954	0.997
	T vs. C	1.211 (1.035–1.417)	78.8	0.996	0.944	0.998
PB	TT vs.CC	1.379 (1.040–1.828)	72.7	0.721	0.972	0.997
	TT vs. CC+CT	1.295 (1.078–1.555)	51.6	0.942	0.856	0.993
	T vs. C	1.181 (1.014–1.376)	78.3	0.999	0.970	0.999
Healthy	TT vs.CC	1.808 (1.252–2.610)	70.4	0.159	0.908	0.972
	TT vs. CC+CT	1.432 (1.207–1.697)	26.1	0.704	0.046	0.598
	CT+TT vs. CC	1.413 (1.047–1.909)	78.4	0.651	0.974	0.997
	T vs. C	1.319 (1.093–1.591)	75.6	0.911	0.806	0.990
Non-gastric cancer	T vs. C	1.138 (1.002–1.291)	77.4	1.000	0.978	0.999
Tumor Location	T vs. C	1.142 (1.022–1.275)	46.8	1.000	0.948	0.998
Cardia						
Histologic subtype	TT vs.CC	1.732 (1.211–2.475)	0.0	0.215	0.923	0.981
Intestinal type	TT vs. CC+CT	1.410 (1.027–1.935)	0.0	0.649	0.981	0.998
Diffuse type	TT vs.CC	1.478 (1.023–2.135)	19.4	0.531	0.986	0.998
*MTHFR A1298C*						
HB	CC vs. AA	0.755 (0.574–0.991)	22.0	0.815	0.981	0.998
	CC vs. AA+AC	0.741 (0.571–0.963)	0.0	0.785	0.969	0.998

Abbreviations: HB, hospital-based studies, PB, population-based studies.

### TSA results

**Figure 5:**
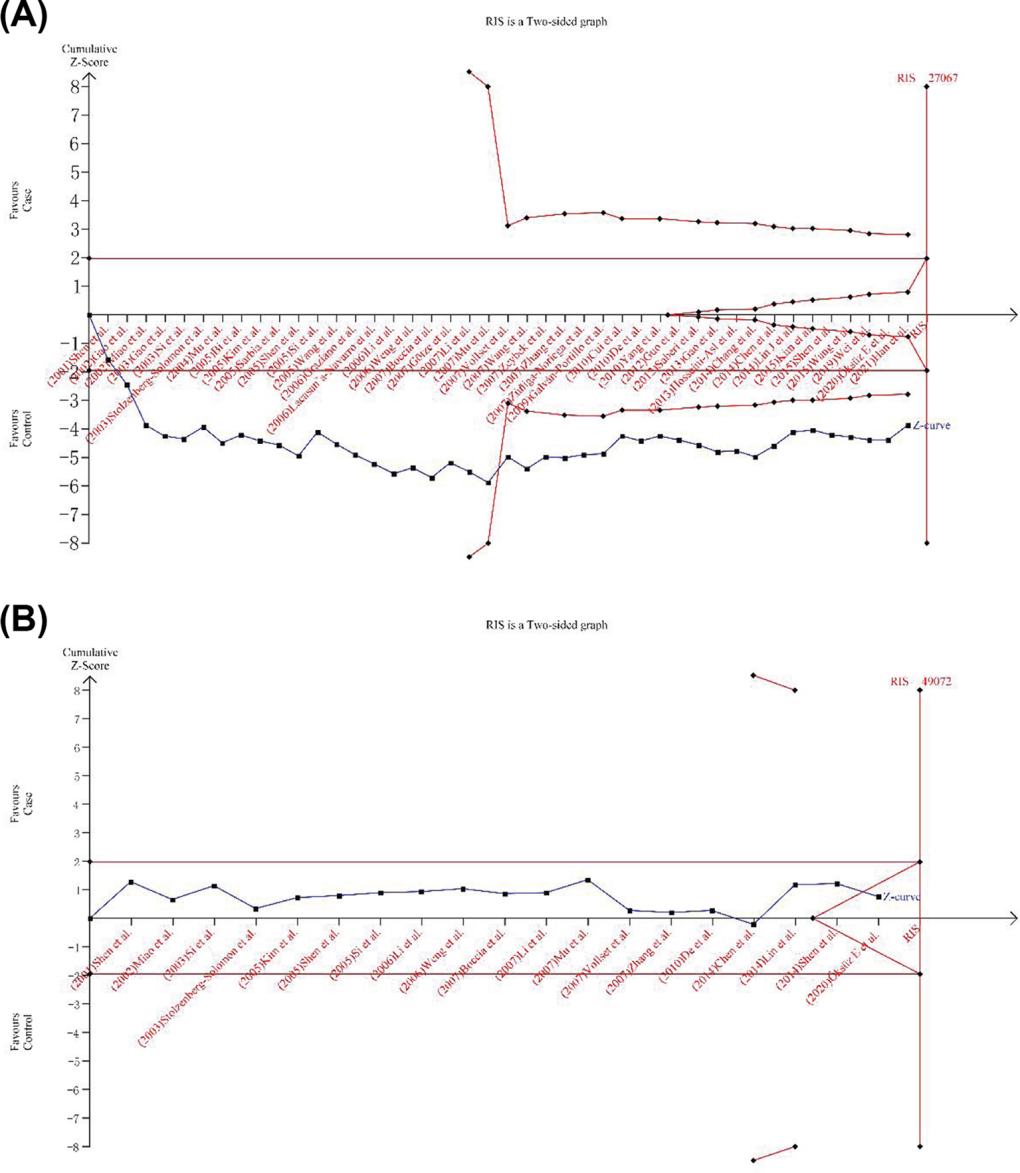
TSA results (**A**) MTHFR C677T(rs1801133); TT vs. CC+CT. (**B**) MTHFR A1298C (rs1801131); CC vs.AA+CC

To reduce random errors and enhance the robustness of the conclusions, we performed TSA (Figure 5). For *MTHFR C677T*, the results show that the curve in the figure crosses both the traditional bound TSA bound, and although the cumulative amount of information does not reach the expected value (RIS), no more tests are needed to confirm the adverse effect of the allele, and positive results are obtained in advance. For *MTHFR A1298C*, the curve in the graph does not cross the traditional threshold and does not cross the TSA threshold, and its cumulative information does not reach the expected information size (TIS), indicating that the traditional meta-analysis may have yielded a false-positive conclusion and that more trials should be included to confirm the effect of the gene.

## Discussion

GC is a malignant tumor originating from the epithelium of the gastric mucosa, which is highly aggressive and heterogeneous [73], and its etiology involves various factors such as smoking, alcohol consumption, pylori infection, immune disorders, and genetic factors [24]. Epidemiological studies are increasingly demonstrating that GC is the result of environmental [17] contextual and genetic interactions; and yet, there is ample evidence that individual susceptibility to cancer development may differ even when exposed to the same environmental carcinogens [24], suggesting that differences exist in population susceptibility to GC and that individual genetic factors play a crucial role in GC [17]. Of note, SNPs in PAR-1, NOD1, NOD2, and DCC genes have been identified to modify GC risk across races [24], and yet polymorphisms in folate-related genes are inconclusive in relation to susceptibility to GC. Some baseline experiments have reported that supplementation with [74] folic acid lowered the rates of GC in mice infected with *Helicobacter pylori*, mainly by enhancing DNA methylation and dampening the inflammatory response, proposed that it may be possible that folate metabolism plays an essential role in malignancy development and progression. More detailed explanation was given in another study: Altered activity of folate metabolizing enzymes or insufficient intake of folate can lead to DNA hypomethylation, which affects DNA synthesis and consequently DNA stability and the expression of proto-oncogenes and oncogenes, which are closely related to tumor development [75]. Extensive studies have been done over recent years on the genetic variation of the *MTHFR* gene to clarify its role that is involved in the etiology of GC. *MTHFR*, one of the key enzymes in folate metabolism, converts 5,10-methylenetetrahydrofolate to 5-methyltetrahydrofolate, which is implicated in purine and pyrimidine synthesis, DNA repair, and broad methylation reactions *in vivo* [17]. The proper functioning of this metabolic pathway is essential in maintaining normal methylation of DNA, *de novo* synthesis of nucleotides, and repair of DNA [17]. By affecting the expression of oncogenes and oncogenes, as well as the stability of the genome, DNA methylation is involved in tumorigenesis and development. Notwithstanding, many studies have attempted to explore the association between *MTHFR* polymorphisms and GC risk. However, it is unfortunate that solid evidence was not obtained, which may be due to different reasons, on account of small sample size, ethnicity and regional differences. Trying to transcend these shortcomings, meta-analysis is a valid option.

Supplementary Table S1 shows the characteristics of this study compared to previous meta-analyses, and as you can see, the number of studies included in this meta-analysis far exceeds the number of published meta-analyses, with a total of 43 studies examining the association between *MTHFR* gene polymorphisms and GC risk [9,30–71], among which 34 studies on the *MTHFR C677T* polymorphism and 19 studies on the *MTHFR A1298C*. Notably, the maximum sample sizes in studies exploring the association between *MTHFR C677T* and *A1298C* polymorphisms and susceptibility to GC were 27 and 12, respectively, compared with published meta-analyses. Furthermore, the latest years of previous meta-analyses on *MTHFR C677T* and *A1298C* polymorphisms and GC susceptibility were 2017 [17] and 2014 [19], respectively, and most of the studies included in the published meta-analyses focused on studies before 2014, whereas the latest study [71] we included was 2021. Reviewing past studies, Shen et al. [30] first investigated the association between *MTHFR* gene polymorphisms and GC in 2001 and showed its possible association with GC risk in a Chinese Han population. In 2006, Zintzaras et al. [28] and Larsson et al. [29] reviewed previous studies on *MTHFR* polymorphism and GC susceptibility by two meta-analyses conducted a comprehensive assessment and showed that: the *MTHFR C677T* polymorphism has a positive association with the occurrence of GC, mainly in Asian populations. This has since been confirmed by several meta-analysis studies [8,17,18,22–27]. Notably, additional meta-analyses [19–21] have suggested that *MTHFR C677T* polymorphism may also be a risk factor for GC in Caucasians. Compared with the results of these meta-analyses, the majority of results were consistent with our results that the *MTHFR C677T* polymorphism increased susceptibility to GC in Asian and Caucasian populations in particular, indicating good stability of our study. Regarding the *MTHFR A1298C* polymorphism, most studies showed no association with GC susceptibility, however, two studies [19,24] showed what appears to be a protective effect, which is consistent with the results of our subgroup analysis based on hospital sources, but no definitive conclusions can be drawn about the association between *MTHFR A1298C* and GC susceptibility because of the lack of stability of the sensitivity analysis results and the fact that confidence assessment and TSA analysis suggest that this result is less reliable.

After confidence assessment, our results showed no significant association between the *MTHFR C677T* and *A1298C* polymorphisms and GC risk. We need to take a dialectical view of this issue, which may be due to the variability in sample sources and study protocols between studies, and more comprehensive and detailed studies are still needed in the future to further explore the relationship between *MTHFR* polymorphisms and GC susceptibility.

Carefully reviewing past published meta-analyses on the *MTHFR C677T* polymorphism and the risk of GC, we found some shortcomings. First of all, Only two meta-analyses [17,26] provided a quality assessment of the included articles, others [8,18–25,27–29] failed, which led to low-quality studies being included in these meta-analyses. Secondly, HWE was not calculated in some of the previous meta-analyses [22,23,29], HWE needs to be used for sound genetic association studies. Selection bias or genotyping errors may exist if controls do not satisfy HWE, and it can lead to misleading results. Furthermore, the genetic models developed were inconsistent between studies. Only 3 articles [19,21,24] out of 14 meta analyses compared five genetic models separately, which may have contributed to false negative results. Finally, statistically significant associations in all previous meta-analyses were not assessed for probabilities of false positive reports [8,17–29]. Thus, it is possible that the results of their meta-analysis are not credible.

In this study, compared with previous meta-analyses, our study had the following strengths: (1) quality assessment and HWE tests were performed for all included studies; (2) a significantly larger sample size in this study than in previous meta-analyses, and more detailed and comprehensive data were gathered, which could avoid errors due to small sample size to some extent; (3) reliability of the data was tested using FPRP, BFDP tests and Venice criteria, which made the study results more rigorous; (4) according to control type, data source, tumor site and histological subtype, further subgroup analysis was performed to enable a deeper understanding of the clinical characteristics of gastric cancer; (5) the sources of heterogeneity were explored using meta-regression analysis.

There are however some limitations of our meta-analysis. (1) Only accepted studies published in English or Chinese, which may have resulted in publication bias by omitting nonsignificant or negative results in other languages, leading to non-detection even using Begg’s funnel plot and Egger’s test. (2) There were no specific analyses for Asian populations, i.e., more detailed results may be obtained from specific analyses for East, West, South, North, Central, and Southeast Asian populations. (3) Controls in some of these studies were selected from non-cancerous patients who underwent gastroscopy, whereas controls in others were selected only from asymptomatic individuals, which leading to misclassification bias due to failure to exclude potential cancer cases in controls. (4) We did not control for confounding factors, such as smoking, alcohol consumption, folic acid intake, family history, age, sex, and variable study design, all of which were strongly associated with influencing the results. Notably, owing to the lack of sufficient data, gene–gene and gene–environment interactions were not fully elucidated in this meta-analysis. (5) Our study found that the association between *MTHFR C677T* and *A1298C* polymorphisms and susceptibility to gastric cancer was variable across races, which may be due to genetic heterogeneity and geographical differences, and with this in mind, future mRNA expression analysis based on genotype could further investigate whether the biological results are consistent with our observed association.

## Conclusion and future perspective

This study further investigated whether the *C677T* and *A1298C* polymorphisms of the *MTHFR* gene were associated with GC risk on the basis of a meta-analysis of existing case-control studies and cohort studies to provide a reference for population-based GC risk assessment, prevention and control, and diagnosis, with the aim of providing new ideas for the prevention and treatment of GC patients. In spite of some limitations and in agreement with several previous studies, the present meta-analysis leads to a strong conclusion that the *MTHFR C677T* polymorphism is significantly associated with GC susceptibility and is a vulnerability factor in Caucasians and Asians, especially in Asian populations, and is also positively associated with cardia-type, intestinal-type GC and diffuse GC. On the contrary, the *MTHFR A1298C* polymorphism may be a protective factor for GC.Taken together, it is suggested that *MTHFR* may be engaged in the etiology of tumorigenesis and its potential relevant therapeutic value in cancer prevention.However, confidence assessment and TSA analysis showed no significant correlation between *MTHFR C677T* and *A1298C* and susceptibility to GC in the context of the current study. A further multicenter, larger sample size, well-designed study, including gene environment interaction assessment, is necessary to confirm our findings.

## Materials and methods

### Search strategy

The search and inclusion of this meta-analysis strictly followed the PRISMA criteria [78]. We retrieved relevant papers from PubMed, EMBASE, and the Chinese Wan fang Data Knowledge System and identified them by screening titles, abstracts, and complete articles. Specifically, this search strategy was applied: (‘polymorphism’ OR ‘variant’ OR ‘variation’ OR ‘mutation’ OR ‘SNP’ OR ‘genome-wide association study’ OR ‘genetic association study’ OR ‘genotype’ OR ‘allele’) AND (‘gastric’ OR ‘stomach’) AND (*MTHFR* OR Methylenetetrahydrofolate reductase OR 5, 10-Methylenetetrahydrofolate reductase). February 2023 is the deadline for the search. We also checked the references of identified meta-analyses and reviews to see if there were other relevant studies.

### Selection criteria

Below are the inclusion criteria: (1) Studies based on case–controls or cohorts; (2) studies examining the association between the polymorphisms in *MTHFR C677T* and *A1298C* and the risk of GC; (3) Literature selected for the case and control groups contains sufficient genotype data. Exclusion criteria included: (1) Study duplications; (2) Data-deficient studies; (3) Meta-analyses, reviews, letters and case reports.

### Data extraction and quality score assessment

Extracted and cross-checked by two investigators on the basis of established inclusion and exclusion criteria. Upon objection, a consensus could not be reached after discussion and negotiation. The corresponding author will be responsible for re-extraction of the data, and then confirming and verifying it. In cases where data is insufficiently detailed or uncertain, try contacting the original author to supplement and confirm the accuracy of the data. The studies with incomplete data were eliminated, and only the best quality studies were retained among the studies with repeated publications, duplications or similar data, and the rest were excluded. As follows is the extracted information The first author's surname, publication year, country, ethnicity ‘Caucasian’, ‘African’, ‘Asian’, ‘Indian’ and ‘mixed population’), and the number of cases and controls, matching variables, and data source Cases and controls’ genotype distributions. Additional details are available in attached Tables 2a and 2b.

### Quality assessment

Drawing on two previous meta-analyses [79,80], we developed quality assessment criteria, as shown in Supplementary Table S4, where two independent authors independently assessed the quality of the extracted data. The corresponding authors were scored again if any disagreement existed. The quality scores of the studies varied from 0 (lowest) to 18 (highest). Studies scoring less than 9 were labeled as low-quality studies, while studies scoring 9–12 were categorized as moderate quality studies and those scoring >12 were defined as high-quality studies.

### Statistical analysis and reliability analysis

By calculating the pooled odds ratios (ORs) with corresponding 95% confidence intervals (95% CIs) for each genetic model gene frequency, *P*<0.05 was seen as statistically meaningful, we assessed the association of *MTHFRC677T* and *A1298C* polymorphisms with GC risk. Five genetic model comparisons we used: (1) allele model (*C677T*: T allele vs. C allele; *A1298C*: C allele vs. A allele); (2) additive model (*C677T*: TT vs. CC; *A1298C*: CC vs. AA); (3) dominant model [*C677T*: (TT + CT) vs. CC; *A1298C*: (CC + AC) vs. AA]; (4) recessive model (*C677T*: TT vs. (CT + CC); *A1298C*: CC vs. (AC + AA); (5) over-dominant model (*C677T*: TT vs. CT; *A1298C*: AC vs. CC).We used Chi-square based *Q*-test and *I*-square test for heterogeneity to assess whether heterogeneity exists between the included literature. If *P*>0.10 and/or *I*-square ≤ 50%, we considered no significant heterogeneity among studies [81] and pooled ORs to apply a fixed effects model (FEM) [82]. If not, the random effects model (REM) was chosen [83] and used meta-regression analysis to explore sources of heterogeneity. Taking into account the potential reasons for the heterogeneity between studies could be ethnicity, tumors site, staging, source of control and type of control, subgroup analysis was run in terms of different races (Caucasian/Asian/mixed/Indian), tumors site (cardia/non-cardia) and tissue type (intestinal/diffuse), source of control (hospital/population), and type of control (healthy/non-cancerous).Moreover, Hardy–Weinberg equilibrium (HWE) was calculated using Chi-square goodness-of-fit test, *P*>0.05 which were defined as HWE, otherwise Hardy–Weinberg disequilibrium (HWD) in control groups. Only high-quality and HWE-compliant studies were used in sensitivity analyses based on the quality score results and HWE conditions. Sensitivity analyses were made through the following three methods: (1) by sequentially excluding one study, (2) by excluding low- and moderate-quality or HWD studies, and (3) by retaining only high-quality and HWE studies. In the meantime, the false positive reporting probability (FPRP) test [84] and the Venice criteria [85], we applied to assess the confidence of statistically significant associations. We performed both Begg’s funnel plot [86] and Egger’s test to estimate the presence of publication bias risk in the selected studies [87]. All statistical analyses described above were performed using STATA 12.0 (Stata Corp LP, College Station, Texas).

### Trial sequential analysis

The trial sequential analysis (TSA) is performed as we previously described [88], in short, we used TSA to reduce random errors and the required information size (RIS) for prediction [89,90]. For this study, TSA was performed using TSA 0.9.5.10 Beta software with operational settings choosing fixed effects (*MTHFR C677T*)/random effects model (*MTHFR A1298C*) as in the previous meta-analysis [91], and type I error probability α = 0.05 and type II error probability β = 0.2 were set, and the accruing information size (AIS) was used to identify the amount of information, the combined effect size was used as OR, and the loss function was the O’Brien-Fleming function, and TSA was performed on the outcome indicator efficiency. Strong evidence is available for our study if the cumulative z-curve passes the monitoring boundary, the RIS line, or enters the useless zone. Otherwise, more research is needed [92].

## Supplementary Material

Supplementary Tables S1-S4

## Data Availability

All data have been included in the article and in the attached tables. Supplementary Table S1 clarifies in detail the details of the studies included in this and previously published meta-analyses; Supplementary Tables S2 and 3 summarise the detailed data for all genotypes of MTHFR C677T and CA1298C; Supplementary Table S4 shows the scoring criteria for quality scoring of all included studies; Supplementary Table S5 provides a summary of the guidelines for preferred re-reporting items for the meta-analysis.

## Abbreviations

95% CI: 95% confidence interval
AIS: accruing information size
BFDP: Bayesian false discovery probability
FEM: Fixed effects model
FPRP: false-positive report probabilities
GC: gastric cancer
HWD: Hardy–Weinberg Disequilibrium
HWE: Hardy–Weinberg equilibrium
*MTHFR*: methylenetetrahydrofolate reductase
OR: odds ratio
PRISMA: Preferred Reporting Items for Systematic Review and Meta-Analyses
REM: random effects model
RIS: required information size
TSA: Trial sequential analysis
