# Supplementary material for: Methylenetetrahydrofolate reductase C677T and A1298C polymorphisms and gastric cancer susceptibility: an updated meta-analysis
Source: Biosci Rep. 2023 Apr 19;43(4):BSR20222553. doi: 10.1042/BSR20222553 (PMC10116338; doi:10.1042/BSR20222553)
Supplement: Supplementary Tables S1-S4 [file BSR-2022-2553_supp.zip › BSR-2022-2553_tableST2.docx]

**Supplemental Table 2.Genotype distribution of MTHFR polymorphisms in the included studies of gastric cancer.**

| **No.** | **First author/Year** | **Country** | **Geographic region** | **Ethnicity** | **Sample size** | **SC** | **Type of control** | **Matching** | **Genotypes distribution of *MTHFR C677T(rs1801133)*** | | | | | | **HWE** | **Quality score** | **Genotypes distribution of *A1298C (rs1801131)*** | | | | | | **HWE** | **Quality score** |  |
| --- | --- | --- | --- | --- | --- | --- | --- | --- | --- | --- | --- | --- | --- | --- | --- | --- | --- | --- | --- | --- | --- | --- | --- | --- | --- |
|  |  |  |  |  |  |  |  |  | **Cases** | | | **Controls** | | |  |  | **Cases** | | | **Controls** | | |  |  |  |
|  |  |  |  |  |  |  |  |  | **CC** | **CT** | **TT** | **CC** | **CT** | **TT** |  |  | **AA** | **AC** | **CC** | **AA** | **AC** | **CC** |  |  |  |
| **1** | **Shen et al.[30] 2001** | **Jiangsu(China)** | **Asia** | **Asian** | **187/166** | **HB** | **Non-gastric cancer Controls** | **Age and sex** | **55** | **90** | **42** | **60** | **80** | **26** | **0.9378** | **16** | **130** | **55** | **2** | **111** | **50** | **5** | **0.8251** | **16** |  |
| **2** | **Gao et al.[31] 2002** | **Jiangsu(China)** | **Asia** | **Asian** | **107/200** | **HB** | **Non-gastric cancer Controls** | **Age** | **22** | **61** | **24** | **63** | **99** | **38** | **0.9356** | **13** | **–** | **–** | **–** | **–** | **–** | **–** | **–** | **–** |  |
| **3** | **Miao et al.[32] 2002** | **Beijing(China)** | **Asia** | **Asian** | **217/468** | **HB** | **Non-gastric cancer Controls** | **Age and sex** | **47** | **107** | **63** | **151** | **217** | **100** | **0.1833** | **17** | **150** | **64** | **3** | **324** | **139** | **5** | **0.0178** | **15** |  |
| **4** | **Stolzenberg-Solomon et al.[33] 2003** | **Linxian(China)** | **Asia** | **Asian** | **309/398** | **PB** | **Non-gastric cancer Controls** | **Age and sex** | **57** | **130** | **122** | **65** | **209** | **124** | **0.1407** | **18** | **232** | **90** | **3** | **294** | **104** | **0** | **0.0027** | **16** |  |
| **5** | **Si et al.[34] 2003** | **Chongqing(China)** | **Asia** | **Asian** | **66/101** | **HB** | **Non-gastric cancer Controls** | **NR** | **30** | **27** | **9** | **49** | **43** | **9** | **0.9209** | **10** | **38** | **27** | **1** | **58** | **38** | **5** | **0.6994** | **10** |  |
| **6** | **Gao et al.[35] 2003** | **China** | **Asia** | **Asian** | **105/200** | **HB** | **Non-gastric cancer Controls** | **Age and sex** | **21** | **60** | **24** | **63** | **99** | **38** | **0.9356** | **14** | **–** | **–** | **–** | **–** | **–** | **–** | **–** | **–** |  |
| **7** | **Mu et al.[36] 2004** | **Jiangsu(China)** | **Asia** | **Asian** | **194/391** | **PB** | **Healthy controls** | **Age and sex** | **50** | **106** | **38** | **135** | **199** | **57** | **0.2348** | **13** | **–** | **–** | **–** | **–** | **–** | **–** | **–** | **–** |  |
| **8** | **Shen et al.[37] 2005** | **Jiangsu(China)** | **Asia** | **Asian** | **320/313** | **PB** | **Non-gastric cancer Controls** | **Age and sex** | **105** | **171** | **44** | **113** | **172** | **28** | **0.001** | **12** | **219** | **93** | **8** | **230** | **73** | **10** | **0.1671** | **16** |  |
| **9** | **Wang et al.[38] 2005** | **Henan(China)** | **Asia** | **Asian** | **129/315** | **PB** | **Non-gastric cancer Controls** | **Age and sex** | **25** | **45** | **59** | **74** | **143** | **98** | **0.1236** | **16** | **–** | **–** | **–** | **–** | **–** | **–** |  | **–** |  |
| **10** | **Sarbia et al.[39] 2005** | **Germany** | **Europe** | **Caucasian** | **213/255** | **HB** | **Healthy controls** | **NR** | **73** | **108** | **32** | **107** | **115** | **33** | **0.8095** | **10** | **–** | **–** | **–** | **–** | **–** | **–** |  | **–** |  |
| **11** | **Si et al.[40] 2005** | **Chongqing(China)** | **Asia** | **Asian** | **122/101** | **HB** | **Healthy controls** | **Age and sex** | **5** | **48** | **16** | **49** | **43** | **9** | **0.9209** | **13** | **73** | **44** | **5** | **58** | **38** | **5** | **0.6994** | **13** |  |
| **12** | **Kim et al.[41] 2005** | **South Korea** | **Asia** | **Asian** | **133/445** | **PB** | **Non-gastric cancer Controls** | **Age and sex** | **42** | **64** | **27** | **143** | **239** | **63** | **0.0203** | **10** | **98** | **34** | **1** | **308** | **129** | **8** | **0.1852** | **14** |  |
| **13** | **Bi et al.[42] 2005** | **Fujian(China)** | **Asia** | **Asian** | **309/188** | **HB** | **Non-gastric cancer Controls** | **Age and sex** | **140** | **149** | **20** | **97** | **76** | **15** | **0.9832** | **15** | **–** | **–** | **–** | **–** | **–** | **–** |  | **–** |  |
| **14** | **Weng et al.[43] 2006** | **Shanghai(China)** | **Asia** | **Asian** | **38/34** | **HB** | **Non-gastric cancer Controls** | **Age and sex** | **14** | **19** | **5** | **15** | **11** | **8** | **0.0586** | **10** | **26** | **12** | **0** | **22** | **11** | **1** | **0.7878** | **10** |  |
| **15** | **Li et al.[44] 2006** | **Jiangsu(China)** | **Asia** | **Asian** | **170/140** | **HB** | **Healthy controls** | **Age and sex** | **61** | **78** | **31** | **67** | **56** | **17** | **0.3258** | **12** | **126** | **42** | **2** | **97** | **41** | **2** | **0.3113** | **12** |  |
| **16** | **Graziano et al.[45] 2006** | **Italy** | **Europe** | **Caucasian** | **162/164** | **PB** | **Non-gastric cancer Controls** | **Age and sex** | **34** | **86** | **42** | **67** | **68** | **29** | **0.1132** | **16** | **–** | **–** | **–** | **–** | **–** | **–** | **–** | **–** |  |
| **17** | **Lacasan ˜a-Navarro et al.[46] 2006** | **Mexican** | **North America** | **Caucasian** | **201/427** | **HB** | **Non-gastric cancer Controls** | **Age and sex** | **56** | **85** | **60** | **144** | **179** | **104** | **0.0014** | **10** | **–** | **–** | **–** | **–** | **–** | **–** | **–** | **–** |  |
| **18** | **Boccia et al.[47] 2007** | **Italy** | **Europe** | **Caucasian** | **102/254** | **HB** | **Non-gastric cancer Controls** | **Age and sex** | **29** | **51** | **22** | **98** | **115** | **41** | **0.4589** | **15** | **50** | **43** | **9** | **125** | **107** | **22** | **0.8944** | **15** |  |
| **19** | **Zhang et al.[48] 2007** | **Poland** | **Europe** | **Caucasian** | **305/427** | **PB** | **Healthy controls** | **Age and sex** | **146** | **116** | **33** | **185** | **178** | **36** | **0.4621** | **17** | **135** | **125** | **31** | **180** | **179** | **41** | **0.7200** | **17** |  |
| **20** | **Mu et al.[49] 2007** | **Jiangsu(China)** | **Asia** | **Asian** | **194/391，196/394** | **PB** | **Healthy controls** | **Age and sex** | **50** | **106** | **38** | **135** | **199** | **57** | **0.2348** | **15** | **147** | **49** | **0** | **275** | **112** | **7** | **0.2490** | **15** |  |
| **21** | **Vollset et al.[50] 2007** | **Mixed（EPIC）** | **Europe** | **Caucasian** | **245/619，244/614** | **PB** | **Non-gastric cancer Controls** | **Age and sex** | **109** | **104** | **32** | **248** | **277** | **94** | **0.2529** | **15** | **103** | **116** | **25** | **315** | **246** | **53** | **0.6147** | **15** |  |
| **22** | **Zeybek et al.[51] 2007** | **Turkey** | **Asia** | **Caucasian** | **35/144** | **HB** | **Non-gastric cancer Controls** | **Age and sex** | **18** | **5** | **12** | **64** | **15** | **65** | **0.0000** | **5** | **–** | **–** | **–** | **–** | **–** | **–** | **–** | **–** |  |
| **23** | **Wang Y et al.[52] 2007** | **Hebei(China)** | **Asia** | **Asian** | **467/540** | **HB** | **Healthy controls** | **Age and sex** | **74** | **203** | **190** | **119** | **234** | **187** | **0.0055** | **13** | **–** | **–** | **–** | **–** | **–** | **–** | **–** | **–** |  |
| **24** | **Li S et al.[53] 2007** | **Nanjing(China)** | **Asia** | **Asian** | **170/140** | **HB** | **Non-gastric cancer Controls** | **Age and sex** | **61** | **78** | **31** | **67** | **56** | **17** | **0.3258** | **12** | **126** | **42** | **2** | **97** | **41** | **2** | **0.3113** | **12** |  |
| **25** | **Zúñiga-Noriega et al.[54] 2007** | **Mexican** | **North America** | **Caucasian** | **51/67** | **HB** | **Non-gastric cancer Controls** | **Age** | **16** | **23** | **12** | **15** | **38** | **14** | **0.2706** | **8** | **–** | **–** | **–** | **–** | **–** | **–** | **–** | **–** |  |
| **26** | **Götze et al.[55] 2007** | **Germany** | **Europe** | **Caucasian** | **103/106** | **HB** | **Non-gastric cancer Controls** | **Age and sex** | **46** | **45** | **12** | **41** | **49** | **16** | **0.8287** | **15** | **–** | **–** | **–** | **–** | **–** | **–** | **–** | **–** |  |
| **27** | **Galván-Portillo et al.[56] 2009** | **Mexican** | **North America** | **Caucasian** | **248/478** | **HB** | **Non-gastric cancer Controls** | **Age and sex** | **37** | **132** | **79** | **89** | **217** | **172** | **0.1629** | **14** | **–** | **–** | **–** | **–** | **–** | **–** | **–** | **–** |  |
| **28** | **De Re et al.[57] 2010** | **Italy** | **Europe** | **Caucasian** | **57/454** | **HB** | **Healthy controls** | **Age and sex** | **18** | **25** | **14** | **152** | **238** | **64** | **0.0568** | **13** | **25** | **27** | **5** | **179** | **234** | **41** | **0.0038** | **11** |  |
| **29** | **Cui LH et al.[58] 2010** | **Korea** | **Asia** | **Asian** | **2213/1700** | **PB** | **Non-gastric cancer Controls** | **NR** | **778** | **1052** | **382** | **540** | **863** | **297** | **0.1326** | **13** | **–** | **–** | **–** | **–** | **–** | **–** | **–** | **–** |  |
| **30** | **Yang et al.[59] 2010** | **Jiangxi(China)** | **Asia** | **Asian** | **139/165** | **HB** | **Healthy controls** | **NR** | **44** | **80** | **15** | **62** | **75** | **28** | **0.5157** | **11** | **–** | **–** | **–** | **–** | **–** | **–** | **–** | **–** |  |
| **31** | **Saberi et al.[60] 2012** | **Iranian** | **Asia** | **Caucasian** | **450/780** | **HB** | **Non-gastric cancer Controls** | **NR** | **198** | **172** | **35** | **422** | **308** | **50** | **0.5341** | **13** | **–** | **–** | **–** | **–** | **–** | **–** | **–** | **–** |  |
| **32** | **Guo et al.[61] 2012** | **Heilongjiang(China)** | **Asia** | **Asian** | **97/104** | **HB** | **Healthy controls** | **Age and sex** | **22** | **48** | **27** | **32** | **57** | **25** | **0.9678** | **12** | **–** | **–** | **–** | **–** | **–** | **–** | **–** | **–** |  |
| **33** | **Gao et al.[62] 2013** | **Neimenggu（China）** | **Asia** | **Asian** | **264/535** | **HB** | **Healthy controls** | **Age and sex** | **115** | **105** | **44** | **277** | **207** | **51** | **0.1791** | **16** | **–** | **–** | **–** | **–** | **–** | **–** | **–** | **–** |  |
| **34** | **Hosseini-Asl SS et al.[63] 2013** | **Iranian** | **Asia** | **Caucasian** | **76/91** | **NR** | **Healthy controls** | **Age and sex** | **47** | **25** | **4** | **41** | **46** | **4** | **0.0439** | **5** | **–** | **–** | **–** | **–** | **–** | **–** | **–** | **–** |  |
| **35** | **Lin J et al.[9] 2014** | **Shentou（China）** | **Asia** | **Asian** | **285/570** | **HB** | **Healthy controls** | **Age and sex** | **146** | **97** | **42** | **248** | **203** | **119** | **0.0000** | **14** | **136** | **115** | **34** | **225** | **235** | **110** | **0.0008** | **16** |  |
| **36** | **Chen J et al.[64] 2014** | **Chengdu(China)** | **Asia** | **Asian** | **767/775** | **PB** | **Healthy controls** | **Age and sex** | **390** | **289** | **88** | **362** | **307** | **105** | **0.0026** | **13** | **316** | **359** | **92** | **331** | **358** | **86** | **0.4615** | **17** |  |
| **37** | **Chang S-C et al.[65] 2014** | **Jiangsu(China)** | **Asia** | **Asian** | **194/391** | **PB** | **Healthy controls** | **Age and sex** | **50** | **106** | **38** | **135** | **199** | **57** | **0.2348** | **16** | **–** | **–** | **–** | **–** | **–** | **–** | **–** | **–** |  |
| **38** | **Wang YF et al.[66] 2015** | **Jiangsu(China)** | **Asia** | **Asian** | **324/592** | **HB** | **Non-gastric cancer Controls** | **Age and sex** | **102** | **148** | **74** | **170** | **318** | **104** | **0.0326** | **10** | **–** | **–** | **–** | **–** | **–** | **–** | **–** | **–** |  |
| **40** | **Kim W et al.[67] 2015** | **Korea** | **Asia** | **Asian** | **271/542** | **HB** | **Healthy controls** | **Age and sex** | **88** | **138** | **45** | **168** | **281** | **92** | **0.1647** | **15** | **–** | **–** | **–** | **–** | **–** | **–** | **–** | **–** |  |
| **41** | **Shen et al.[68] 2015** | **Tianjin(China)** | **Asia** | **Asian** | **160/160** | **HB** | **Healthy controls** | **Age and sex** | **72** | **64** | **24** | **78** | **69** | **13** | **0.6765** | **10** | **58** | **84** | **18** | **62** | **77** | **21** | **0.703** | **10** |  |
| **42** | **Wei L et al.[69] 2019** | **Xian(China)** | **Asia** | **Asian** | **679/756** | **HB** | **Healthy controls** | **Age and sex** | **192** | **327** | **160** | **234** | **361** | **161** | **0.3225** | **12** | **–** | **–** | **–** | **–** | **–** | **–** | **–** | **–** |  |
| **43** | **Öksüz E et al.[70] 2020** | **Turkey** | **Asia** | **Caucasian** | **70/61** | **HB** | **Healthy controls** | **Age and sex** | **39** | **21** | **10** | **36** | **16** | **9** | **0.0066** | **4** | **18** | **35** | **17** | **34** | **16** | **11** | **0.0024** | **6** |  |
| **43** | **Han Z, Sheng H [ 71] 2021** | **Jiangsu(China)** | **Asia** | **Asian** | **307/560** | **HB** | **Non-gastric cancer Controls** | **Age and sex** | **127** | **140** | **40** | **164** | **295** | **101** | **0.1124** | **15** | **–** | **–** | **–** | **–** | **–** | **–** | **–** | **–** |  |

**(HB：hospital-based study, PB：population-based study, NR：not reported,NA：not available)**
