# Supplementary material for: Methylenetetrahydrofolate reductase C677T and A1298C polymorphisms and gastric cancer susceptibility: an updated meta-analysis
Source: Biosci Rep. 2023 Apr 19;43(4):BSR20222553. doi: 10.1042/BSR20222553 (PMC10116338; doi:10.1042/BSR20222553)
Supplement: Supplementary Tables S1-S4 [file BSR-2022-2553_supp.zip › BSR-2022-2553_tableST1.doc]

**Supplemental Table 1.** **Included studies of MTHFR polymorphisms in gastric cancer within the meta-analyses (A, Asian; C, Caucasian).**

| **No.** | **First Author/Year** | **Country** | **Eligible research studies of *MTHFR C677T (rs1801133)*** | | | | | | | | | | | | | | **Eligible research studies of *MTHFR A1298C (rs1801131)*** | | | | | | | |
| --- | --- | --- | --- | --- | --- | --- | --- | --- | --- | --- | --- | --- | --- | --- | --- | --- | --- | --- | --- | --- | --- | --- | --- | --- |
|  |  |  | **All studies** | | | | | | | | | | | | | | **All studies** | | | | | | | |
|  |  |  | **This study** | **Wang et al.**  **[17] 2017** | **Xu**  **et al.**  **[18] 2016** | **Xia**  **et al.**  **[19] 2014** | **Tang et al.**  **[20] 2014** | **Yan S et al. [21] 2014** | **Chen et al. [22] 2014** | **Sun et al.**  **[23] 2014** | **Lv L et al.**  **[24] 2013** | **Dong et al.**  **[25] 2010** | **Sun L et al.**  **[8] 2008** | **Sun L et al.**  **[26] 2008** | **Boccia**  **et al.**  **[27] 2007** | **Zintzaras et al. [28] 2006** | **This study** | **Xia**  **et al.**  **[19] 2014** | **Dong et al.**  **[25] 2010** | **Sun L et al.**  **[8] 2008** | **Sun L et al.**  **[26] 2008** | **Boccia et al.**  **[27] 2007** | **Zintzaras et al.**  **[28] 2006** | **Larsson SC**  **et al. [29] 2006** |
| **1** | **Shen et al.[30] 2001** | **Jiangsu(China)** | **A** | **A** | **–** | **A** | **–** | **–** | **–** | **A** | **–** | **A** | **–** | **A** | **–** | **–** | **A** | **–** | **A** | **A** | **–** | **A** | **–** | **–** |
| **2** | **Gao et al.[31] 2002** | **Jiangsu(China)** | **A** | **A** | **A** | **A** | **–** | **A** | **A** | **–** | **A** | **A** | **A** | **A** | **A** | **–** | **–** | **–** | **–** | **–** | **–** | **–** | **–** | **–** |
| **3** | **Miao et al.[32] 2002** | **Beijing(China)** | **A** | **A** | **A** | **A** | **A** | **A** | **A** | **A** | **A** | **A** | **A** | **A** | **A** | **A** | **A** | **A** | **A** | **A** | **A** | **A** | **A** | **A** |
| **4** | **Stolzenberg-Solomon et al.[33] 2003** | **Linxian(China)** | **A** | **–** | **A** | **A** | **A** | **A** | **A** | **–** | **A** | **A** | **A** | **A** | **A** | **A** | **A** | **A** | **A** | **A** | **A** | **A** | **–** | **A** |
| **5** | **Si et al.[34] 2003** | **Chongqing(China)** | **A** | **A** | **–** | **–** | **–** | **–** | **–** | **–** | **–** | **–** | **–** | **–** | **–** | **–** | **A** | **–** | **–** | **–** | **–** | **–** | **–** | **–** |
| **6** | **Gao et al.[35] 2003** | **China** | **A** | **–** | **–** | **–** | **–** | **–** | **–** | **–** | **A** | **–** | **–** | **–** | **A** | **–** | **–** | **–** | **–** | **–** | **–** | **–** | **–** | **–** |
| **7** | **Mu et al.[36] 2004** | **Jiangsu(China)** | **A** | **A** | **–** | **–** | **–** | **–** | **–** | **A** | **–** | **–** | **–** | **A** | **A** | **–** | **–** | **–** | **–** | **–** | **–** | **–** | **–** | **–** |
| **8** | **Shen et al.[37] 2005** | **Jiangsu(China)** | **A** | **A** | **A** | **–** | **A** | **A** | **A** | **A** | **A** | **–** | **A** | **A** | **–** | **A** | **A** | **A** | **–** | **–** | **A** | **A** | **A** | **A** |
| **9** | **Wang et al.[38] 2005** | **Henan(China)** | **A** | **–** | **A** | **A** | **A** | **A** | **A** | **A** | **A** | **A** | **A** | **–** | **A** | **A** | **–** | **–** | **–** | **–** | **–** | **–** | **–** | **–** |
| **10** | **Sarbia et al.[39] 2005** | **Germany** | **C** | **–** | **C** | **C** | **C** | **C** | **C** | **–** | **C** | **C** | **–** | **C** | **C** | **–** | **–** | **–** | **–** | **–** | **–** | **–** | **–** | **–** |
| **11** | **Si et al.[40] 2005** | **Chongqing(China)** | **A** | **–** | **A** | **A** | **–** | **A** | **A** | **A** | **A** | **A** | **A** | **–** | **A** | **–** | **A** | **–** | **A** | **A** | **A** | **–** | **A** | **–** |
| **12** | **Kim et al.[41] 2005** | **South Korea** | **A** | **–** | **A** | **A** | **A** | **A** | **A** | **A** | **A** | **A** | **–** | **A** | **A** | **A** | **A** | **A** | **A** | **A** | **–** | **A** | **A** | **A** |
| **13** | **Bi et al.[42] 2005** | **Fujian(China)** | **A** | **A** | **–** | **A** | **–** | **A** | **A** | **A** | **A** | **A** | **A** | **A** | **–** | **–** | **–** | **–** | **–** | **–** | **–** | **–** | **–** | **–** |
| **14** | **Weng et al.[43] 2006** | **Shanghai(China)** | **A** | **A** | **A** | **A** | **A** | **A** | **A** | **A** | **A** | **A** | **A** | **A** | **A** | **–** | **A** | **–** | **A** | **A** | **A** | **A** | **A** | **–** |
| **15** | **Li et al.[44] 2006** | **Jiangsu(China)** | **A** | **–** | **–** | **A** | **–** | **–** | **A** | **A** | **–** | **–** | **–** | **–** | **–** | **–** | **A** | **–** | **A** | **–** | **–** | **–** | **–** | **–** |
| **16** | **Graziano et al.[45] 2006** | **Italy** | **C** | **–** | **C** | **C** | **C** | **C** | **C** | **C** | **C** | **C** | **–** | **C** | **C** | **C** | **–** | **C** | **–** | **–** | **–** | **–** | **–** | **–** |
| **17** | **Lacasan ˜a-Navarro et al.[46] 2006** | **Mexican** | **C** | **–** | **C** | **–** | **C** | **C** | **C** | **C** | **C** | **C** | **–** | **–** | **C** | **C** | **–** | **C** | **–** | **–** | **–** | **–** | **–** | **–** |
| **18** | **Boccia et al.[47] 2007** | **Italy** | **C** | **–** | **C** | **C** | **C** | **C** | **C** | **C** | **C** | **C** | **–** | **–** | **C** | **–** | **C** | **–** | **C** | **C** | **C** | **–** | **C** | **–** |
| **19** | **Zhang et al.[48] 2007** | **Poland** | **C** | **–** | **C** | **C** | **–** | **C** | **C** | **–** | **C** | **C** | **–** | **C** | **C** | **–** | **C** | **–** | **C** | **C** | **–** | **C** | **C** | **–** |
| **20** | **Mu et al.[49] 2007** | **Jiangsu(China)** | **A** | **–** | **–** | **A** | **A** | **A** | **A** | **–** | **A** | **A** | **A** | **–** | **–** | **–** | **A** | **–** | **A** | **A** | **A** | **–** | **–** | **–** |
| **21** | **Vollset et al.[50] 2007** | **Mixed（EPIC）** | **C** | **–** | **C** | **C** | **–** | **C** | **C** | **–** | **C** | **C** | **–** | **–** | **–** | **–** | **C** | **–** | **C** | **C** | **–** | **–** | **–** | **–** |
| **22** | **Zeybek et al.[51] 2007** | **Turkey** | **C** | **–** | **C** | **C** | **C** | **C** | **C** | **C** | **C** | **C** | **–** | **C** | **C** | **–** | **–** | **–** | **–** | **–** | **–** | **–** | **–** | **–** |
| **23** | **Wang Y et al.[52] 2007** | **Hebei(China)** | **A** | **A** | **A** | **–** | **–** | **A** | **A** | **–** | **A** | **A** | **A** | **A** | **A** | **–** | **–** | **–** | **–** | **–** | **–** | **–** | **–** | **–** |
| **24** | **Li S et al.[53] 2007** | **Nanjing(China)** | **A** | **–** | **A** | **–** | **–** | **A** | **A** | **–** | **A** | **A** | **A** | **–** | **–** | **–** | **A** | **–** | **–** | **A** | **A** | **–** | **–** | **–** |
| **25** | **Zúñiga-Noriega et al.[54] 2007** | **Mexican** | **C** | **–** | **C** | **–** | **–** | **C** | **C** | **C** | **C** | **C** | **–** | **–** | **–** | **–** | **–** | **–** | **–** | **–** | **–** | **–** | **–** | **–** |
| **26** | **Götze et al.[55] 2007** | **Germany** | **C** | **–** | **C** | **C** | **–** | **C** | **C** | **C** | **C** | **C** | **–** | **–** | **C** | **–** | **–** | **–** | **–** | **–** | **–** | **–** | **–** | **–** |
| **27** | **Galván-Portillo et al.[56] 2009** | **Mexican** | **C** | **–** | **C** | **C** | **C** | **C** | **C** | **–** | **C** | **C** | **–** | **–** | **–** | **–** | **–** | **–** | **–** | **–** | **–** | **–** | **–** | **–** |
| **28** | **De Re et al.[57] 2010** | **Italy** | **C** | **–** | **C** | **C** | **C** | **C** | **C** | **–** | **C** | **–** | **–** | **–** | **–** | **–** | **C** | **–** | **C** | **–** | **–** | **–** | **–** | **–** |
| **29** | **Cui LH et al.[58] 2010** | **Korea** | **A** | **–** | **A** | **A** | **–** | **A** | **–** | **A** | **A** | **–** | **–** | **–** | **–** | **–** | **–** | **–** | **–** | **–** | **–** | **–** | **–** | **–** |
| **30** | **Yang et al.[59] 2010** | **Jiangxi(China)** | **A** | **A** | **–** | **A** | **–** | **–** | **A** | **A** | **–** | **–** | **–** | **–** | **–** | **–** | **–** | **–** | **–** | **–** | **–** | **–** | **–** | **–** |
| **31** | **Saberi et al.[60] 2012** | **Iranian** | **C** | **–** | **C** | **C** | **–** | **C** | **C** | **C** | **C** | **–** | **–** | **–** | **–** | **–** | **–** | **–** | **–** | **–** | **–** | **–** | **–** | **–** |
| **32** | **Guo et al.[61] 2012** | **Heilongjiang(China)** | **A** | **A** | **–** | **A** | **–** | **–** | **–** | **A** | **–** | **–** | **–** | **–** | **–** | **–** | **–** | **–** | **–** | **–** | **–** | **–** | **–** | **–** |
| **33** | **Gao et al.[62] 2013** | **Neimenggu（China）** | **A** | **A** | **–** | **A** | **–** | **–** | **A** | **A** | **–** | **–** | **–** | **–** | **–** | **–** | **–** | **–** | **–** | **–** | **–** | **–** | **–** | **–** |
| **34** | **Hosseini-Asl SS et al.[63] 2013** | **Iranian** | **A** | **–** | **A** | **–** | **–** | **–** | **–** | **–** | **C** | **–** | **–** | **–** | **–** | **–** | **–** | **–** | **–** | **–** | **–** | **–** | **–** | **–** |
| **35** | **Lin J et al.[9] 2014** | **Guangdong（China）** | **A** | **–** | **A** | **–** | **–** | **–** | **–** | **–** | **–** | **–** | **–** | **–** | **–** | **–** | **A** | **–** | **–** | **–** | **–** | **–** | **–** | **–** |
| **36** | **Chen J et al.[64] 2014** | **Chengdu(China)** | **A** | **–** | **A** | **–** | **–** | **–** | **–** | **–** | **–** | **–** | **–** | **–** | **–** | **–** | **A** | **–** | **–** | **–** | **–** | **–** | **–** | **–** |
| **37** | **Chang S-C et al.[65] 2014** | **Jiangsu(China)** | **A** | **–** | **A** | **–** | **–** | **–** | **–** | **–** | **–** | **–** | **–** | **–** | **–** | **–** | **–** | **–** | **–** | **–** | **–** | **–** | **–** | **–** |
| **38** | **Wang YF et al.[66] 2015** | **Jiangsu(China)** | **A** | **–** | **–** | **–** | **–** | **–** | **–** | **–** | **–** | **–** | **–** | **–** | **–** | **–** | **–** | **–** | **–** | **–** | **–** | **–** | **–** | **–** |
| **40** | **Kim W et al.[67] 2015** | **Korea** | **A** | **–** | **–** | **–** | **–** | **–** | **–** | **–** | **–** | **–** | **–** | **–** | **–** | **–** | **–** | **–** | **–** | **–** | **–** | **–** | **–** | **–** |
| **41** | **Shen et al.[68] 2015** | **Tianjin(China)** | **A** | **–** | **–** | **–** | **–** | **–** | **–** | **–** | **–** | **–** | **–** | **–** | **–** | **–** | **A** | **–** | **–** | **–** | **–** | **–** | **–** | **–** |
| **42** | **Wei L et al.[69] 2019** | **Xian(China)** | **A** | **–** | **–** | **–** | **–** | **–** | **–** | **–** | **–** | **–** | **–** | **–** | **–** | **–** | **–** | **–** | **–** | **–** | **–** | **–** | **–** | **–** |
| **43** | **Öksüz E et al.[70] 2020** | **Turkey** | **C** | **–** | **–** | **–** | **–** | **–** | **–** | **–** | **–** | **–** | **–** | **–** | **–** | **–** | **C** | **–** | **–** | **–** | **–** | **–** | **–** | **–** |
| **43** | **Han et al. [71] 2021** | **Jiangsu(China)** | **A** | **–** | **–** | **–** | **–** | **–** | **–** | **–** | **–** | **–** | **–** | **–** | **–** | **–** | **–** | **–** | **–** | **–** | **–** | **–** | **–** | **–** |
