# Supplementary material for: Methylenetetrahydrofolate reductase C677T and A1298C polymorphisms and gastric cancer susceptibility: an updated meta-analysis
Source: Biosci Rep. 2023 Apr 19;43(4):BSR20222553. doi: 10.1042/BSR20222553 (PMC10116338; doi:10.1042/BSR20222553)
Supplement: Supplementary Tables S1-S4 [file BSR-2022-2553_supp.zip › BSR-2022-2553_tableST3.doc]

**Supplemental Table 3.Genotypes distribution of *MTHFR C677T (rs1801133)* by Histologic subtype.**

| **No.** | **First author/Year** | **Histologic subtype** | **Genotypes distribution of MTHFR C677T (rs1801133)** | | | | | |
| --- | --- | --- | --- | --- | --- | --- | --- | --- |
|  |  |  | **Cases** | | | **Controls** | | |
|  |  |  | **CC** | **CT** | **TT** | **CC** | **CT** | **TT** |
| **Histologic subtype** | **Sarbia et al.[39] 2005** | **Intestinal type** | **69** | **59** | **14** | **422** | **308** | **50** |
|  |  | **Diffuse type** | **39** | **34** | **7** | **422** | **308** | **50** |
|  | **Graziano et al.[45] 2006** | **Intestinal type** | **19** | **47** | **25** | **67** | **68** | **29** |
|  |  | **Diffuse type** | **15** | **39** | **17** | **67** | **68** | **29** |
|  | **Lacasan ˜a-Navarro et al.[46] 2006** | **Intestinal type** | **17** | **32** | **19** | **144** | **179** | **104** |
|  |  | **Diffuse type** | **32** | **44** | **35** | **144** | **179** | **104** |
|  | **Götze et al.[55] 2007** | **Intestinal type** | **21** | **24** | **8** | **41** | **49** | **16** |
|  |  | **Diffuse type** | **18** | **15** | **4** | **41** | **49** | **16** |
|  | **Hosseini-Asl SS et al.[63] 2013** | **Intestinal type** | **28** | **18** | **3** | **41** | **46** | **4** |
|  |  | **Diffuse type** | **19** | **7** | **1** | **41** | **46** | **4** |

**Supplemental Table 3.Genotypes distribution of *MTHFR C677T (rs1801133)* and *A1298C(rs1801131)* by Tumor Location.**

| **No.** | **First author/Year** | **Tumor Location** | **Genotypes distribution of MTHFR C677T (rs1801133)** | | | | | | **Genotypes distribution of MTHFR A1298C (rs1801131)** | | | | | |
| --- | --- | --- | --- | --- | --- | --- | --- | --- | --- | --- | --- | --- | --- | --- |
|  |  |  | **Cases** | | | **Controls** | | | **Cases** | | | **Controls** | | |
|  |  |  | **CC** | **CT** | **TT** | **CC** | **CT** | **TT** | **AA** | **AC** | **CC** | **AA** | **AC** | **CC** |
| **Tumor Location** | **Stolzenberg-Solomon et al.[33] 2003** | **Cardia** | **40** | **94** | **85** | **65** | **209** | **124** | **163** | **53** | **3** | **294** | **104** | **0** |
|  |  | **Non-cardia** | **17** | **36** | **37** | **65** | **209** | **124** | **69** | **21** | **0** | **294** | **104** | **0** |
|  | **Si et al.[34] 2003（a）** | **Cardia** | **11** | **2** | **1** | **49** | **43** | **9** | **8** | **5** | **1** | **58** | **38** | **5** |
|  |  | **Non-cardia** | **19** | **25** | **8** | **49** | **43** | **9** | **30** | **22** | **0** | **58** | **38** | **5** |
|  | **Si et al.[34] 2003（b）** | **Cardia** | **–** | **–** | **–** | **–** | **–** | **–** | **15** | **12** | **2** | **58** | **38** | **5** |
|  |  | **Non-cardia** | **–** | **–** | **–** | **–** | **–** | **–** | **58** | **32** | **3** | **58** | **38** | **5** |
|  | **Shen et al.[37] 2005** | **Cardia** | **42** | **60** | **23** | **113** | **172** | **28** | **98** | **26** | **1** | **230** | **73** | **10** |
|  |  | **Non-cardia** | **63** | **111** | **21** | **113** | **172** | **28** | **121** | **67** | **7** | **230** | **73** | **10** |
|  | **Sarbia et al.[39] 2005** | **Cardia** | **77** | **60** | **15** | **422** | **308** | **50** | **–** | **–** | **–** | **–** | **–** | **–** |
|  |  | **Non-cardia** | **99** | **98** | **13** | **422** | **308** | **50** | **–** | **–** | **–** | **–** | **–** | **–** |
|  | **Si et al.[40] 2005** | **Cardia** | **21** | **71** | **1** | **49** | **43** | **9** | **15** | **12** | **2** | **58** | **38** | **5** |
|  |  | **Non-cardia** | **37** | **41** | **15** | **49** | **43** | **9** | **58** | **32** | **3** | **58** | **38** | **5** |
|  | **Bi et al.[42] 2005** | **Cardia** | **75** | **72** | **8** | **97** | **76** | **15** | **–** | **–** | **–** | **–** | **–** | **–** |
|  |  | **Non-cardia** | **65** | **77** | **12** | **97** | **76** | **15** | **–** | **–** | **–** | **–** | **–** | **–** |
|  | **Graziano et al.[45] 2006** | **Cardia** | **7** | **25** | **11** | **67** | **68** | **29** | **–** | **–** | **–** | **–** | **–** | **–** |
|  |  | **Non-cardia** | **27** | **61** | **31** | **67** | **68** | **29** | **–** | **–** | **–** | **–** | **–** | **–** |
|  | **Götze et al.[55] 2007** | **Cardia** | **13** | **13** | **1** | **41** | **49** | **16** | **–** | **–** | **–** | **–** | **–** | **–** |
|  |  | **Non-cardia** | **33** | **32** | **11** | **41** | **49** | **16** | **–** | **–** | **–** | **–** | **–** | **–** |
|  | **Chen J et al.[64] 2014** | **Cardia** | **77** | **66** | **24** | **362** | **307** | **105** | **–** | **–** | **–** | **–** | **–** | **–** |
|  |  | **Non-cardia** | **313** | **223** | **64** | **362** | **307** | **105** | **–** | **–** | **–** | **–** | **–** | **–** |
