# Supplementary material for: Methylenetetrahydrofolate reductase C677T and A1298C polymorphisms and gastric cancer susceptibility: an updated meta-analysis
Source: Biosci Rep. 2023 Apr 19;43(4):BSR20222553. doi: 10.1042/BSR20222553 (PMC10116338; doi:10.1042/BSR20222553)
Supplement: Supplementary Tables S1-S4 [file BSR-2022-2553_supp.zip › BSR-2022-2553_tableST4.doc]

**Supplemental Table4 Scale for quality assessment of molecular association studies of GC.**

| **Criterion** | **Score** |
| --- | --- |
| **Source of case** | |
| **Selected from population or cancer registry** | **2** |
| **Selected from hospital** | **1** |
| **Not described** | **0** |
| **Source of control** | |
| **Population-based** | **2** |
| **Hospital-based** | **1** |
| **Not described** | **0** |
| **Ascertainment of cancer** | |
| **Histological or pathological confirmation** | **2** |
| **Diagnosis of** **gastric cancer by patient medical record** | **1** |
| **Not described** | **0** |
| **Ascertainment of control** | |
| **Controls were tested to screen out gastric cancer** | **2** |
| **Controls were subjects who did not report gastric cancer, no objective testing** | **1** |
| **Not described** | **0** |
| **Matching** | |
| **Controls matched with cases by age and sex** | **2** |
| **Controls matched with cases only by age or sex** | **1** |
| **Not matched or not described** | **0** |
| **Genotyping examination** | |
| **Genotyping done blindly and quality control** | **2** |
| **Only genotyping done blindly or quality control** | **1** |
| **Unblinded and without quality control** | **0** |
| **HWE** | |
| **HWE in the control group** | **2** |
| **Hardy-Weinberg disequilibrium in the control group** | **0** |
| **Association assessment** | |
| **Assess association between genotypes and gastric cancer with appropriate statistics and adjustment for confounders** | **2** |
| **Assess association between genotypes and gastric cancer with appropriate statistics without adjustment for confounders** | **1** |
| **Inappropriate statistics used** | **0** |
| **Total sample size** |  |
| **≥200** | **2** |
| **<200** | **0** |

**HWE: Hardy-Weinberg equilibrium;GC: gastric cancer**
